# Mechanical activation and expression of HSP27 in epithelial ovarian cancer

**DOI:** 10.1038/s41598-024-52992-7

**Published:** 2024-02-03

**Authors:** Molly Buckley, Maranda Kramer, Bronte Johnson, Gillian Huskin, Joel Berry, Mary Kathryn Sewell-Loftin

**Affiliations:** 1 Department of Biomedical Engineering, University of Alabama at Birmingham, 1824 6thAvenue South, Wallace Tumor Institute, Room 630A, Birmingham, AL 35294 UK; 2 O’Neal Comprehensive Cancer Center, University of Alabama at Birmingham, Birmingham, AL 35233 UK

**Keywords:** Ovarian cancer, Cancer microenvironment

## Abstract

Understanding the complex biomechanical tumor microenvironment (TME) is of critical importance in developing the next generation of anti-cancer treatment strategies. This is especially true in epithelial ovarian cancer (EOC), the deadliest of the gynecologic cancers due to recurrent disease or chemoresistance. However, current models of EOC progression provide little control or ability to monitor how changes in biomechanical parameters alter EOC cell behaviors. In this study, we present a microfluidic device designed to permit biomechanical investigations of the ovarian TME. Using this microtissue system, we describe how biomechanical stimulation in the form of tensile strains upregulate phosphorylation of HSP27, a heat shock protein implicated in ovarian cancer chemoresistance. Furthermore, EOC cells treated with strain demonstrate decreased response to paclitaxel in the in vitro vascularized TME model. The results provide a direct link to biomechanical regulation of HSP27 as a mediator of EOC chemoresistance, possibly explaining the failure of such therapies in some patients. The work presented here lays a foundation to elucidating mechanobiological regulation of EOC progression, including chemoresistance and could provide novel targets for anti-cancer therapeutics.

## Introduction

Ovarian cancer is the deadliest malignancy of all gynecological cancers with a 5-year survival rate of ~ 50%, primarily due to the chemoresistant behaviors of ovarian cancer cells which leads to high rates of recurrence and death^1,2^. The current standard of care for epithelial ovarian cancer (EOC) patients is primary debulking surgery followed by a combination of taxane- and platinum-based chemotherapies^3^. These therapeutic agents target the biochemical signaling that controls cancer growth but not the biomechanical signaling promoting tumor progression. Specifically, these therapies induce hyper- stabilization of cytoskeletal microtubules preventing microtubule shortening or lengthening, ultimately inducing apoptosis in EOC^4^. In spite of the success of these interventions, pathologic biomechanical forces within the tumor microenvironment (TME) of epithelial tumors of all types are emerging as potent regulators of cancer progression; these forces include compression at the tumor interior^5^, tension at the tumor periphery^6^ and altered or insufficient blood flow in the tumor vasculature due to high interstitial shear stresses^7^. In many cases, the pathologic mechanical environment of tumors promotes more invasive behaviors and correlates with worse prognoses^5,6,8,9^. The focus of this study is a deeper understanding of mechanotransduction in EOC, or the cellular behaviors in response to changing biomechanical cues in the TME, which will help us elucidate novel therapeutic strategies and understand limitations in current treatments.

Mechanotransduction is a fundamental process in early human development^10^, stem cell differentiation^11^, and tissue homeostasis^12,13^. The mechanotransmission, mechanosensing, and mechanoresponse of a cell placed under fluctuating mechanical forces results in altered gene expression and cell behavior, including proliferation and migration in both developing and mature healthy microenvironments^14^. However, during tumor progression, cancer cells are able to hijack normal mechanobiological pathways and manipulate the mechanoresponse to allow for cell proliferation, migration, chemoresistance, immunosuppression, and even promote angiogenesis^8,9,12,15–21^. While mechanotransduction studies of all epithelial cancer types have increased in number in the past 20 years, few specifically EOC mechanotransduction studies exist^9,20–26^ and none have previously focused on the effect of tensile stress on EOC chemoresistance. Previous work in our lab demonstrated the profound effects of tensile strain on ovarian cancer cell proliferation, migration, epithelial-to-mesenchyal transition marker expression, and tumor growth in vivo and found that tensile stress increases these phenotypes^22^. However, further investigations are needed to determine the effect tensile strain has on ovarian cancer chemoresponse. To address this and build on our prior results, we developed a highly advanced in vitro model of the vascularized ovarian TME where investigations of EOC behaviors with respect to mechanical stimuliation could be readily quantified. We hypothesized that mechanical stimulation via tensile strain increases stress proteins and that this corresponds with increases in chemoresistance.

Heat shock protein 27 (HSP27) is a small heat shock protein (HSP) of 27 kDa molecular weight^27^. HSP27 is expressed and activated under stress conditions such as high temperatures, hypoxia, and increased shear stress^28–30^. In normal tissues, HSP27 acts as an ATP-independent molecular chaperone that is oligomerized through the WDPF motif when the cells experience heat shock and moves to the nucleus upon activation^31,32^. The molecular chaperoning trait of HSP27 prevents denatured proteins from aggregating and deforming^28,30,33,34^. HSP27 is found to be upregulated in ovarian cancer and has been shown to lead to increased chemoresistance in ovarian cancer cells as a potential off-target or post-target effect due to cisplatin or paclitaxel treatment^35–37^. Additionally, evidence suggests that HSP27 acts a mechanotransducer, polarizing to the front of cells exposed to shear stress^30^. While these studies are a promising start to elucidating the role of HSP27 in ovarian cancer and determining its function as a possible biomarker or therapeutic target, investigating how HSP27 is regulated in EOC through mechanotransduction signaling pathways has not yet been explored. Thus, this study investigated the HSP27 expression in the EOC cells placed under tensile strain in a vascularized 3D microfluidic model of EOC with appropriate matrix components to understand the effect of tensile strain on EOC cell chemoresistance.

## Results

### Optimization of matrix in a 3D vacularized TME model

**Figure 1:**
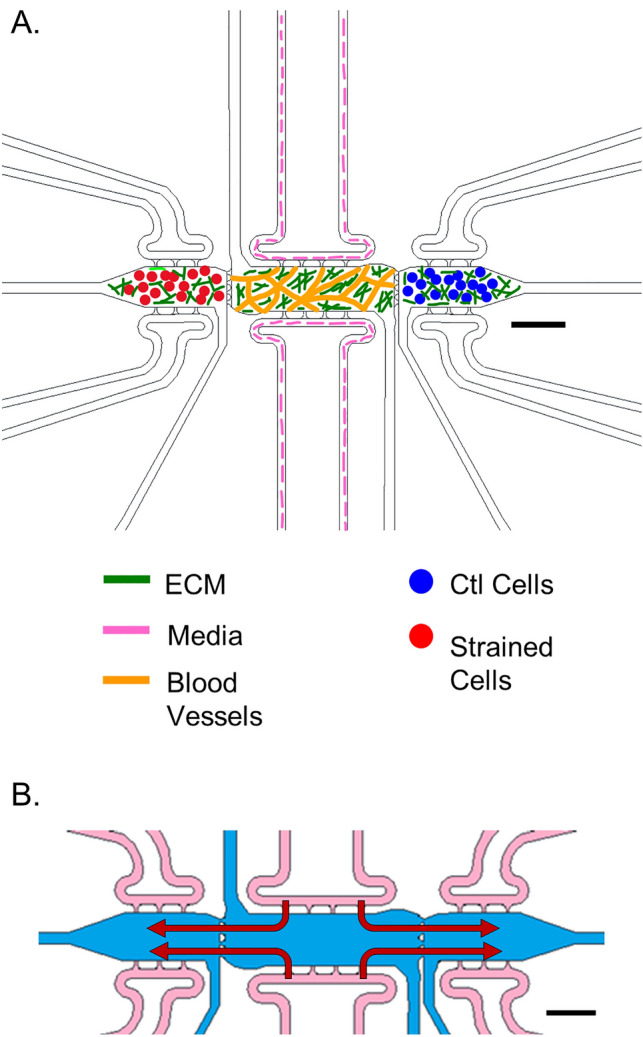
Schematic of 3D multi-microtissue model experimental setup and flow conditions. (**A**) Illustration of typical setup for experiments, showing the 3 microtissues or chamber regions connected in parallel within a microfluidic device. The middle chamber was loaded such that a vascular network of self-assembled HMECs. Side microtissues were loaded with control (non-strained) or strained EOC cells. Each microtissue can be loaded independently, allowing for control over cell spatial orientation. Fluidic lines (top and bottom) allow for control over feeding of cells and diffusion between microtissues. (**B**) Flow pattern for the 3D TME experiments whereby media enters the device through the fluidic lines (pink) attached to the middle chamber (blue) and moves outward to the side chambers (red arrows). Scale bars = 500 μm *Note*—While all chambers have independent fluidic lines, only the media lines for the center chamber were utilized in this study. For more information on flow patterns in this model, see Ref. #49. Portions of this figure were previously published. Reproduced from Ref. 49 with permission from the Royal Society of Chemistry.

**Figure 2:**
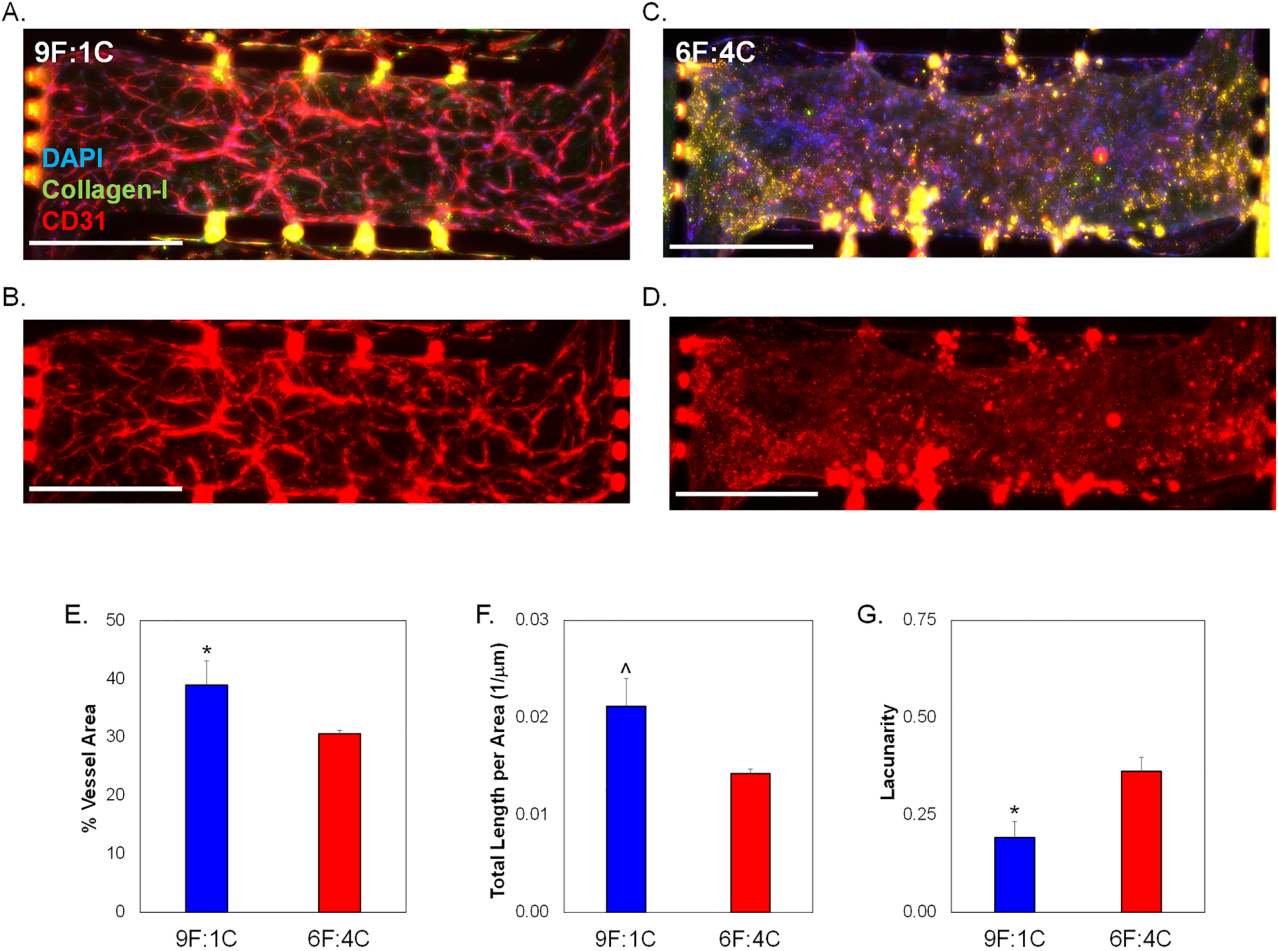
Analysis of microvessel formation in microtissue models testing varying collagen I concentrations. Total ECM content was held constant at 10 mg/mL, while the ratio of fibrin to collagen - was varied. 9 parts fibrin to 1 part collagen is abbreviated 9F:1C; with 6F:4C representing 6 parts fibrin to 4 parts collagen-I. No cancer cells were loaded in side chambers for these experiments, to focus on vascularization potential only of the different matrix combinations. Representative images of center microtissues of devices with (**A**,**B**) 9F:1C and (**C**,**D**) 6F:4C. Immunostaining includes DAPI, CD31 (TXRED), and collagenI (GFP). Scale bars = 200 µm. (**E**) Quantification of average blood vessel area in center chambers. (**F**) Total length of vessels per area in side chambers. (**G**) Average lacunarity of vessels in microtissues. Data shown as average + SEM, N = 3. Samples compared via Mann–Whitney paired tests. *p < 0.05; ^ p = 0.06.

As collagen-I is an important component of the EOC TME^38,39^, we initially studied varying the amount of collagen-I incorporated into 3D microfluidic TME models (Fig. 1). A minimum and maximum ratio of fibrin:collagen was tested while keeping the pH level and total matrix protein concentration (10 mg/mL) the same. A 9:1 ratio of fibrin:collagen was the minimum tested (Fig. 2A,B), while a 6:4 ratio of fibrin:collagen was the maximum ratio tested (Fig. 2C,D). Blood vessel formation was examined via CD31 staining in both matrix configurations. The percent of image with vessels (Fig. 2E), total vessel length per area (Fig. 2F), and mean lacunarity (Fig. 2G), which quantifies the stochasticity or irregularity of the developed vessels, were calculated^40^. The ratio of 9:1 fibrin:collagen samples show a statistically significant ~ 5% increase in vessel percentage area as well as a ~ 50% decrease in patterning or regularity as measured by lower mean lacunarity than the ratio of 6:4 fibrin:collagen. Total vessel length per area was somewhat higher in 9F:1C samples compared to 6F:4C (p = 0.06). All subsequent studies utilized the 9F:1C ratio for matrix composition.

### Strain alters HSP27 phosphorylation and expression

**Figure 3:**
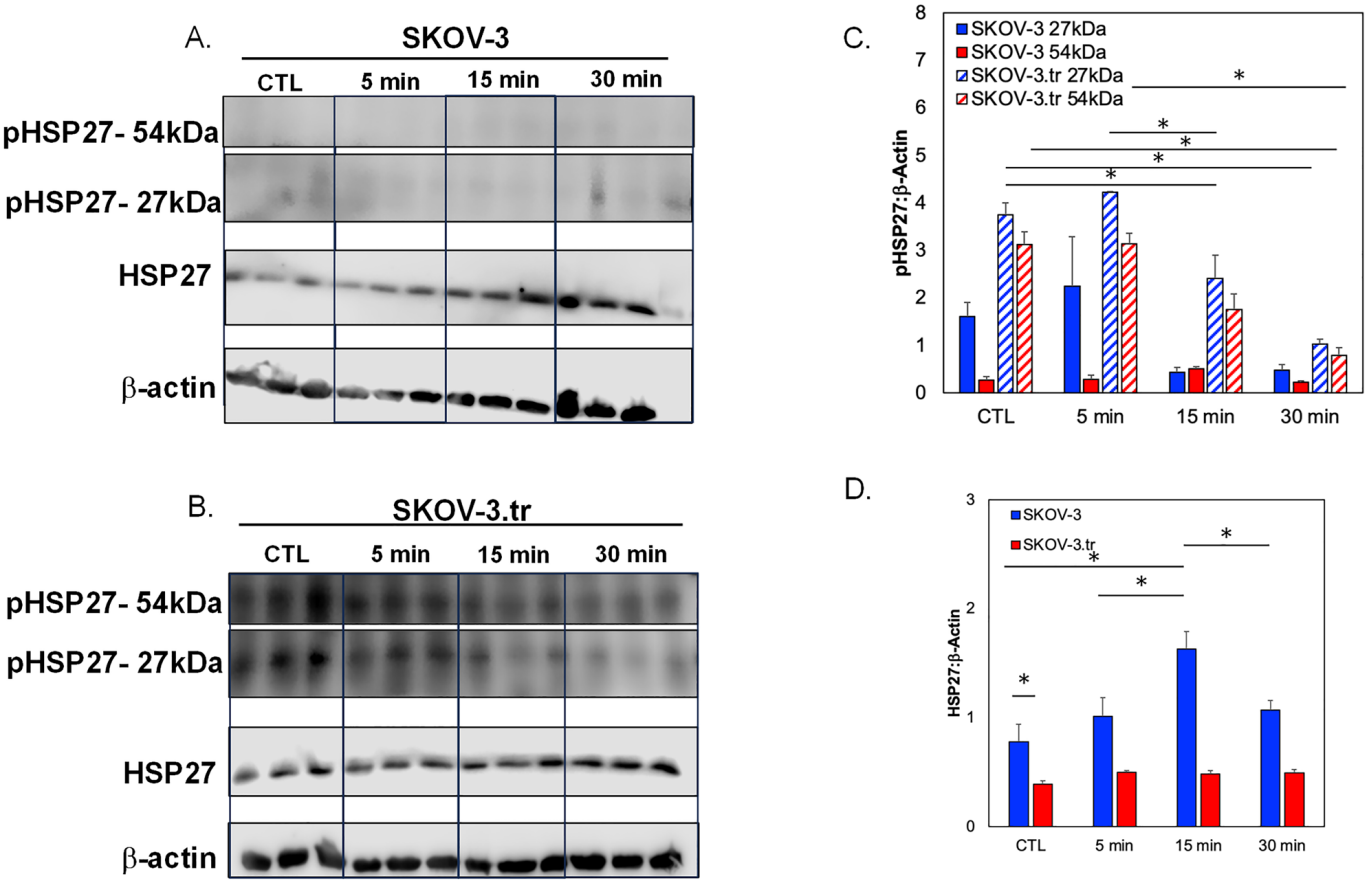
Western blot analysis of pHSP27 and total HSP27 expression in SKOV-3 and SKOV-3.tr cell lines. (**A**) Images of Western blot analysis of pHSP27 and total HSP27 in SKOV-3 cells subjected to varying times of strain stimulation. (**B**) Western blot analysis of pHSP27 and total HSP27 in SKOV-3.tr cells subjected to varying times of strain stimulation. All controls (Ctl) were plated on Flexcell plates for the designated times but were not exposed to strain. (**C**) Quantification of normalized pHSP27 expression in Western blot samples. (**D**) Quantification of normalized total HSP27 expression in Western blot samples. All bands were normalized to β-actin as a loading control. Legend: Solid colors represent SKOV-3 cell samples; striped colors represent SKOV-3.tr samples. Blue is control (Ctl, non-strained) while red is strained samples. Data shown as average + SEM; N = 3 samples. The same experiment was processed on parallel gels/membranes. *p < 0.05. Samples were compared with ANOVA and post-hoc Tukey HSD tests.

**Figure 4:**
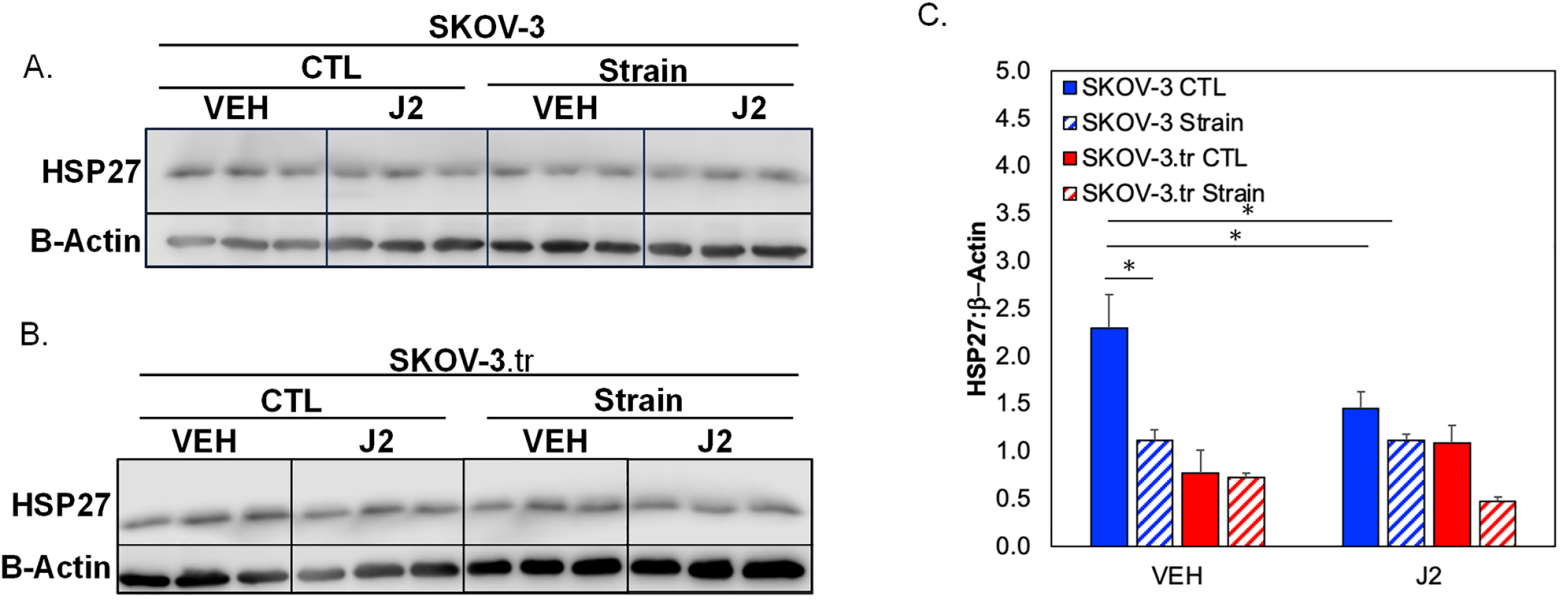
Western blot analysis of total HSP27 expression in SKOV-3 and SKOV-3.tr cell lines with J2 inhibition. (**A**) Images of Western Blot analysis of total HSP27 in SKOV-3 cells subjected to 10 μM J2 treatment and strain stimulation (30 min). (**B**) Images of Western blot analysis of total HSP27 in SKOV-3.tr cells subjected to 10 μM J2 treatment and strain stimulation (30 min). Cropped images shown; full membranes in Supplemental Data section. All controls (CTL) were plated on Flexcell plates but were not exposed to strain. (**C**) Quantification of normalized HSP27 expression Western blot samples; Legend: Blue represents SKOV-3 samples and red represents SKOV-3.tr samples. All bands were normalized to β-actin as a loading control. Data shown as average + SEM; N = 3 samples; *p < 0.05. Samples were compared with ANOVA and post-hoc Tukey HSD tests.

**Figure 5:**
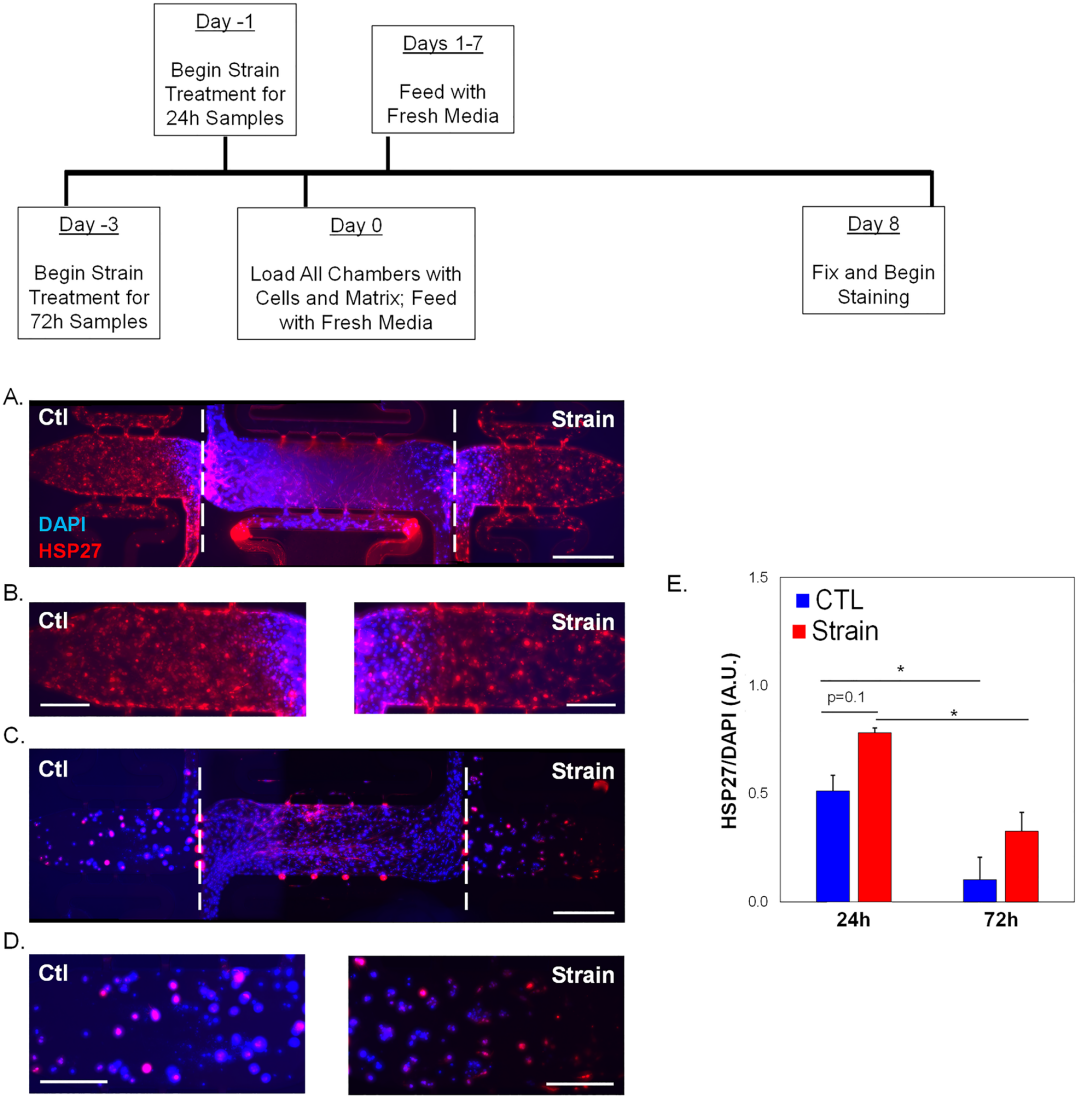
HSP27 expression in 3D TME models with strained SKOV-3 cells. (**A**) Representative immunofluorescence image of full-length 3D microfluidic devices with either control or strained (24 h treatment) SKOV-3 cells in side chambers. (**B**) Side chambers from (**A**). (**C**) Representative immunofluorescence image of full-length 3D microfluidic devices with either control or strained (72 h treatment) SKOV-3 cells in side chambers. (**D**) Side chambers from (**C**). Devices are stained for HSP27 (TXRED) and DAPI. Center chambers are loaded with HMECs and NHLFs. Scale bar = 200 µm (**A**,**C**) or 100 μm (**B**,**D**). White dashed lines show interfaces between microtissue chambers. (**D**) Quantification of HSP27 expression shown in the previous panels. Data shown as average + SEM; N = 3 samples. *p < 0.05. Samples were compared with ANOVA and post-hoc Tukey HSD tests.

Phosphorylation dynamics of HSP27 in response to tensile strain were examined in SKOV-3 (Fig. 3A) or taxol-resistance SKOV-3.tr (Fig. 3B) cell lines. When initially analyzing the effects of tensile strain on SKOV-3, the application of tensile strain shows a small decreases in the monomer pHSP27 (Ser15) levels and no significant changes in the dimer of pHSP27(Fig. 3C). The SKOV-3.tr cell line demonstrates the same trending decreases in the 27 kDa pHSP27 levels and a decrease in the 54 kDa pHSP27 (Fig. 3C). The SKOV-3 cells line exhibits lower levels of both pHSP27 forms compared to SKOV-3.tr cells regardess of strain treatment. When analyzing the total HSP27, increased HSP27 expression was seen in SKOV-3 cells after 15 min before a decrease at longer time periods (Fig. 3D). The SKOV-3.tr cell line shows no change in HSP27 expression with strain for all durations of treatment (Fig. 3D). When analyzing the changes of HSP27 with strain treatment (30 min) in the presence of HSP27 inhibitor, J2 (24 h pre-treatment), it was observed that J2 only decreased HSP27 in the SKOV-3 no strain samples (Fig. 4). The SKOV-3 samples that were treated with strain did not show a decrease in HSP27 expression with the presence of the inhibitor. Likewise, HSP27 expression in neither the control or strained SKOV-3.tr cells n was impacted by the inhibitor (Fig. 4B). In a 3D TME microtissue model, pre-treating SKOV-3 cells with tensile strain increases the expression of HSP27 after both 24h and 72h compared to non-strained (Ctl) cells (Fig. 5). Samples with cells that had been strained for 24 h also had significantly higher HSP27 expression than devices with cells that had been strained for 72 h (Fig. 5D).

### 3D TME models show changes in apoptosis markers for strain-treated cells

**Figure 6:**
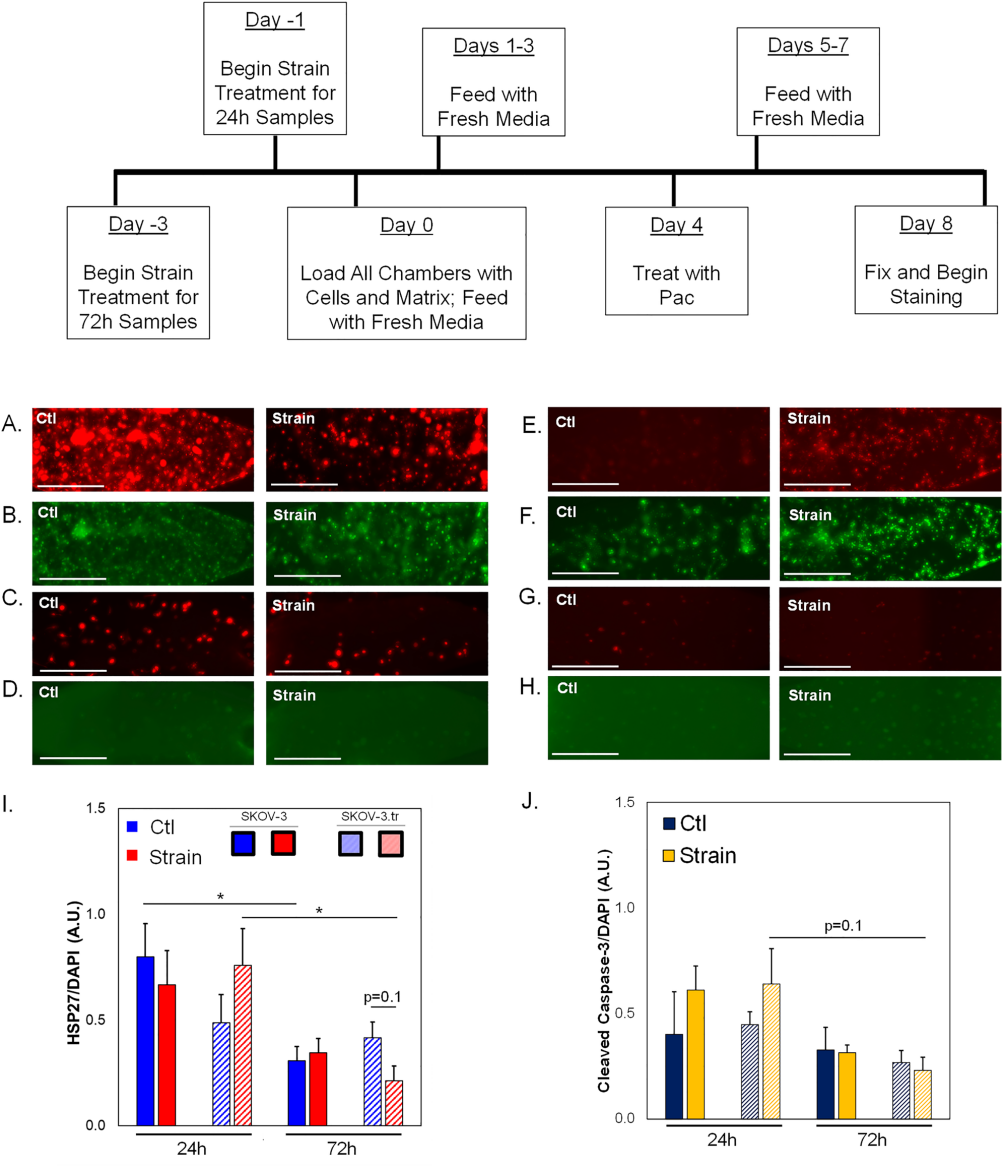
HSP27 and Cleaved Caspase-3 expression in 3D TME models treated with Paclitaxel. (**A**–**H**) Representative fluorescence images of side microtissue regions in devices with either SKOV-3 (**A**–**D**) or SKOV-3.tr (**E**–**H**) cells treated with 24 h (**A**,**B**,**E**,**F**) or 72 h (**C**,**D**,**G**,**H**) of strain compared to control (non-strained) cells. HSP27 immunofluorescence (**A,C,E,G**—TXRED) or Cleaved Caspase-3 (**B**,**D**,**F**,**H**—GFP) (**I**) Quantification of HSP27 expression normalized to DAPI fluorescence. (**A**,**C**,**E**,**G**). (J) Quantification of Cleaved Caspase-3 expression normalized to DAPI fluorescence (**B**,**D**,**F**,**H**). cale bar = 100 µm. Solid color bars are SKOV-3 samples; lines represent SKOV-3.tr samples.Data shown as average + SEM; N = 3 samples; *p < 0.05. HSP27 data was compared with Kruskal-Walls and post-hoc Dunn’s tests; CC-3 data was compared with ANOVA and post-hoc Tukey HSD tests.

**Figure 7:**
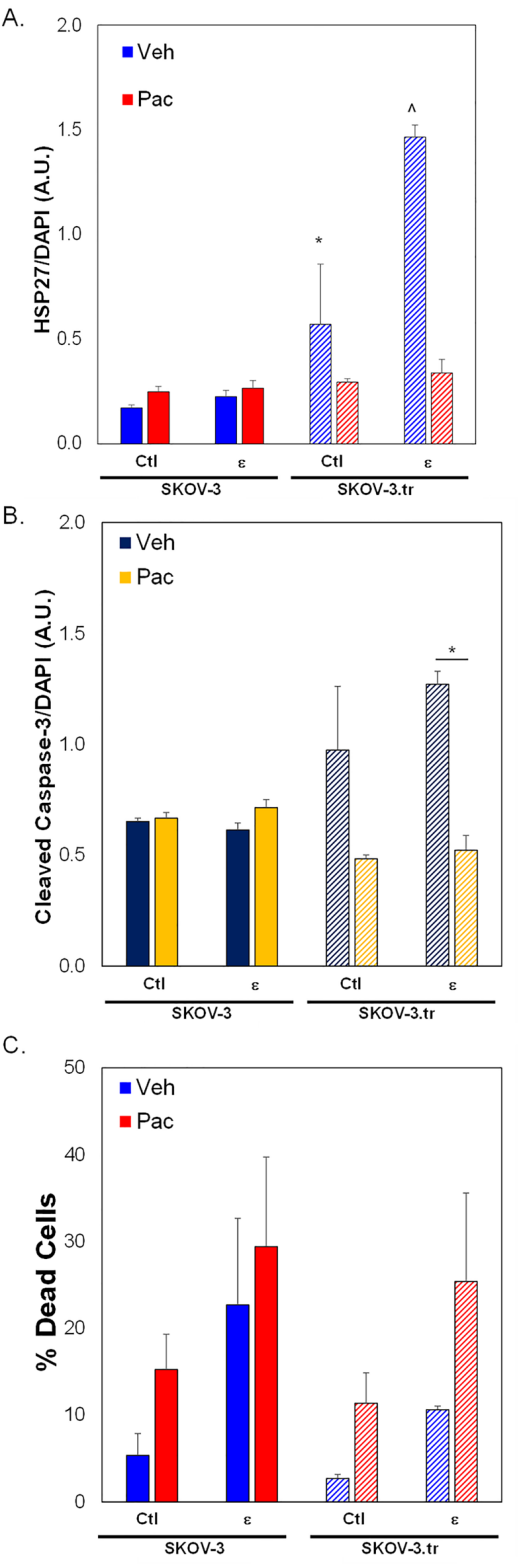
Paclitaxel effect on cells in 2D experiments. SKOV-3 and SKOV-3.tr cells were pre-treated with strain for 24 h, re-plated on tissue culture dishes, and then treated with paclitaxel for 24 h prior to analysis for HSP27 (**A**) or cleaved caspase-3 (**B**). Fluorescence was normalized to DAPI fluorescence. (**C**) Cells pre-treated with 24 h strain followed by paclitaxel treatment were also analyzed via Sytox staining for cell death. Data shown as average + SEM; N = 3 samples. *p < 0.05. Samples were compared with ANOVA and post-hoc Tukey HSD tests.

**Figure 8:**
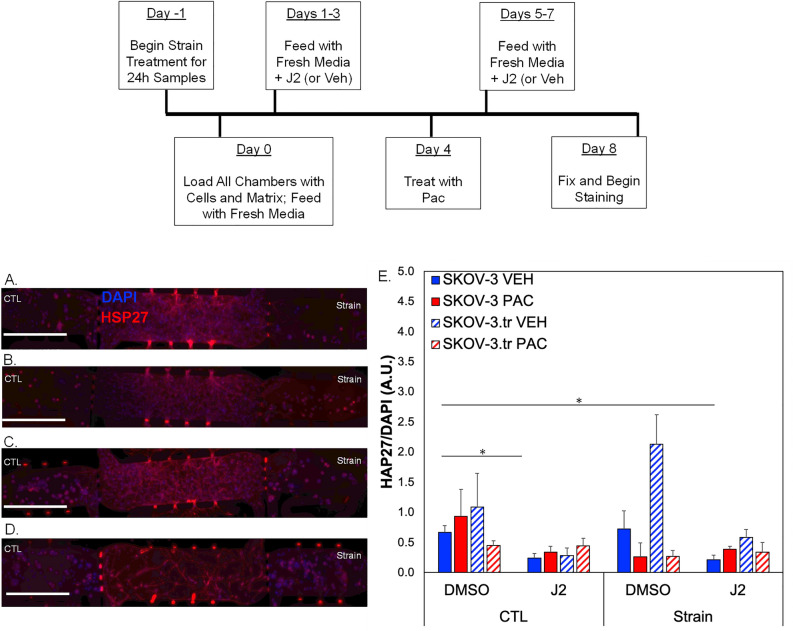
HSP27 expression in 3D TME models pre-treated with J2 and treated with paclitaxel. (**A**–**D**) Representative fluorescence images of microtissue regions in devices with SKOV-3 cells treated with 10 µM of J2 before 24 h of strain compared to control (non-strained) cells. (**E**) Quantification of HSP27 expression normalized to DAPI fluorescence. Data shown as average + SEM; N = 3, *p < 0.1; scale bar = 200 µm. Solid color bars are SKOV-3 samples; lines represent SKOV-3.tr samples. Samples were compared with Kruskal–Wallis tests followed by post-hoc Dunn’s tests.

Microtissues treated with paclitaxel (PAC) show divergent HSP27 responses depending on how long cells were exposed to strain (Fig. 6A–H; Fig. S2). Overall, the results indicate ~ 2X higher expression of HSP27 for SKOV-3 cells and ~ 3X higher expression for SKOV-3.tr cells that are pretreated with 24 h strain compared to 72h strain (Fig. 6I). Additionally, after 72 h of strain treatment, less HSP27 was observed in SKOV-3.tr only cells compared to controls (p = 0.1). No differences were seen between Ctl and strain-treated SKOV-3 cells, although longer strain stimulation somewhat reduced HSP27 levels. On the other hand, the early apoptosis marker cleaved caspase-3 (CC-3) was not significantly different between SKOV-3 cells regardless of strain treatment (Fig. 6J). Levels of CC-3 are significantly reduced in strain-treated SKOV-3.tr cells compared to SKOV-3 cells after 72h of stimulation. There is no difference in cell viability in the devices based on strain pre-treatment (Fig. S1).As there was a significant time delay (4 days) between paclitaxel treatment and analysis in the 3D TME models, we studied the expression of HSP27 and CC-3 in 2D assays on a shorter time scale (~ 24 h post strain treatment). Results show that SKOV-3.tr cells treated with strain and paclitaxel have less HSP27 (Fig. 7A) and less CC-3 (Fig. 7B) compared to strained, vehicle treated controls. As a further control, we completed Sytox analysis for cell death and saw that while paclitaxel did somewhat increase cell death in all samples, even when no large changes in HSP27 or CC-3 were observed (Fig. 7C). When SKOV-3 cells were not strained and treated with J2 in the microtissue model, decreases were observed in HSP27 expression for SKOV-3 and SKOV-3.tr cells control (nonstrained) and VEH (DMSO vehicle control for paclitaxel) (Fig. 8E). In the SKOV-3 cells, the J2 treatment was able to keep HSP27 expression lower in the strain treated cells treated with both VEH and PAC compared to the SKOV-3 cells that were not strained and Veh (DMSO vehicle control for J2) (Fig. 8E). While strain increased HSP27 in SKOV-3.tr cells treated with Veh, J2 blocks this effect for VEH but not PAC treated cells (Fig. 8E).

### HSP27 regulation via strain in additional EOC lines

**Figure 9:**
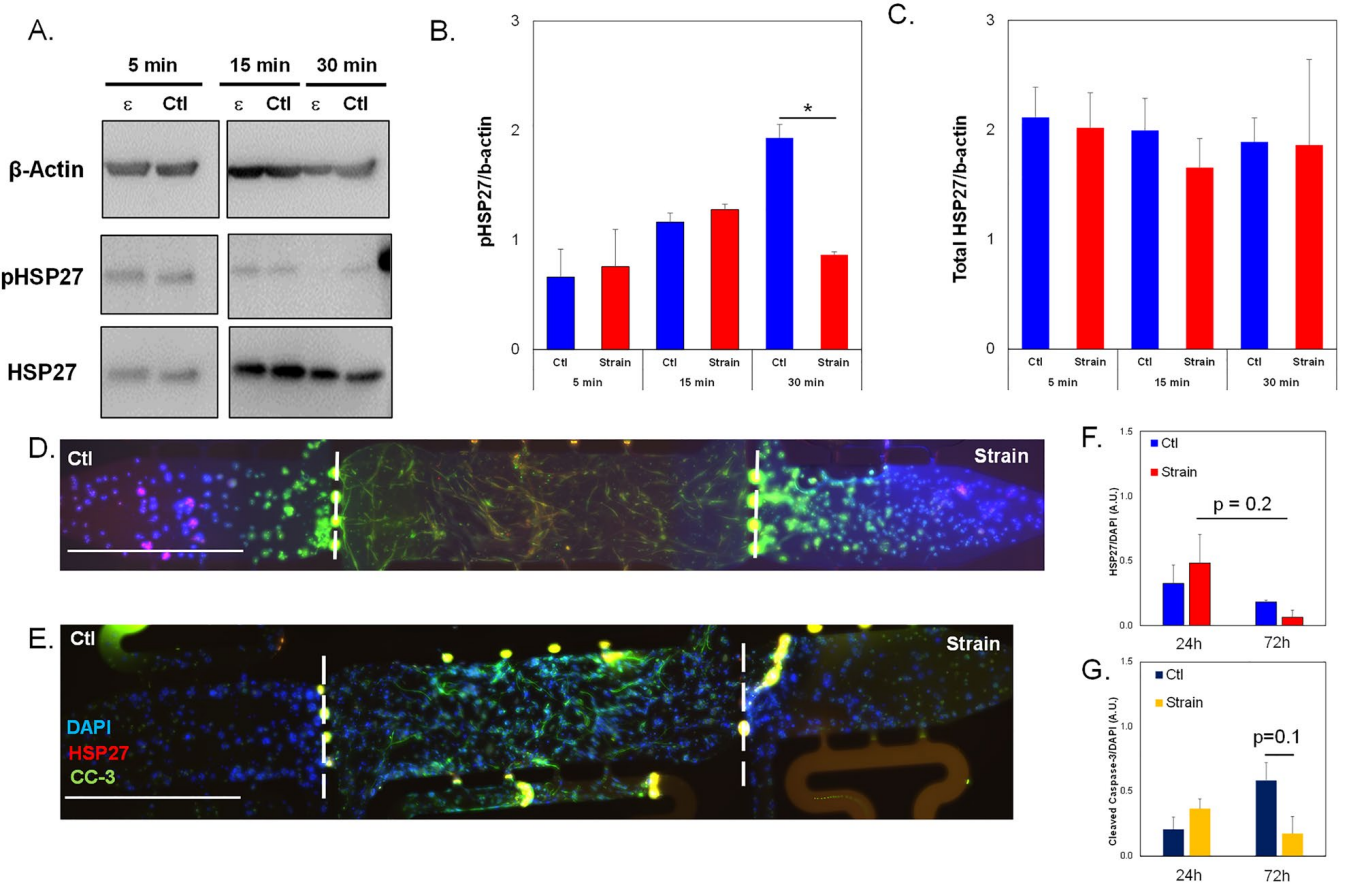
HSP27 expression in OVCAR-8 cells subjected to strain. (**A**) Representative images of Western blot analysis of OVCAR-8 cells subjected to 5, 15 or 30 min of tensile strain compared to control cells with no strain (Ctl). Cropped images shown; full membranes in Supplemental Data section. (**B**) Quantification of pHSP27 expression in Western blot samples normalized to β-actin loading controls. (**C**) Quantification of total HSP27 expression normalized to β-actin loading controls in Western blot samples. (**D**,**E**) Representative fluorescence images of 3D microtissues with OVCAR-8 cells. Devices with OVCAR-8 cells placed under oscillatory tensile strain for (**D**) 24 h vs (**E**) 72 h. Microtissue models were stained for DAPI, HSP27 (TXRED) and cleaved caspase-3 (CC-3, GFP). Scale bar = 200 µm. White dashed lines show interfaces between chambers. (**F**) Quantification of HSP27 expression normalized to DAPI in 3D microtissues side chambers shown in (**D**,**E**). (**G**) Quantification of CC-3 expression normalized to DAPI in 3D microtissue side chambers shown in (**D**,**E**). Data shown as average + SEM; N = 3 samples. *p < 0.05. Samples were compared with ANOVA and post-hoc Tukey HSD tests. The same experiment was processed on parallel gels/membranes.

To determine if these findings were extended to cell lines representative of other histological subtypes of ovarian cancer, these same experiments were performed in OVCAR-8, a serous ovarian cancer cell line. Our results show a nonsignificant increase of ~ 1.6X in pHSP27 in cells strained for 15 min compared to 5 min and a significant decrease of ~ 30% in pHSP27 expression in strained cells from 15 to 30 min. In the 30 min samples, strain significantly reduced pHSP27 levels by ~ 65% compared to non-strained control groups (Fig. 9A,B). There were no significant changes in normalized total HSP27 expression between groups or between timepoints (Fig. 9C). For microtissue studies with non-strained (Ctl) and strained cells, results indicate higher HSP27 levels in the cells that pretreated with 24 h strain compared to non-strained controls and less HSP27 in cells strained for 72 h compared to time-matched controls (Fig. 9D–F). OVCAR-8 cells pretreated with 72 h of strain show a decrease in CC-3 expression compared to non-strained controls as well as to cells strained for only 24h (Fig. 9G).

## Discussion

### Optimization of 3D vascularized ovarian TME models for studying mechanobiology and chemoresponse

Mechanotransduction of physical forces to biochemical signaling has been shown to lead to cancer progression, migration, and even chemoresistance, although the precise mechanisms that cause EOC to become resistant to therapeutics are poorly understood^8,9,17,20,41^. Tensile forces are known to be present along the tumor periphery of a growing tumor, but the magnitude and frequency of this force in ovarian cancer is unknown. Proteins affected by mechanotransduction in cancer through biomechanical signaling include Twist, YAP, MAPK, and ROCK, to name a few^9,42–46^. Downstream of MAPK in the p38 signaling pathway is HSP27, which is shown to be mechanically sensitive^30,47,48^ and has also been shown to be expressed in high amounts in chemoresistant ovarian cancer^36,37^. Moreover, the link between HSP27 and potential off-target effects of paclitaxel may explain why this therapy fails in patients with recurring disease^36^. This study aimed to investigate the relationship between increasing tensile stress in ovarian cancer, activation and expression of HSP27, as well as the chemoresistant nature of EOC. While there are numerous other mechanical stimuli present in the ovarian TME, our studies represent a critical first step in defining the specific role of tensile stress on EOC behaviors in a 3D in vitro model. Future studies can expand to generate more sophisticated models of the EOC TME incorporating tensile strains, shear stress, and matrix stiffness.

A crucial first step of this project focused on optimizing a previously established 3D TME model to be used for ovarian cancer studies^49^. The advantages of the in vitro model over mouse models of ovarian cancer are the control over biomechanical parameters during cell loading and the ability to do imaging in these 3D human microtissue models. To more closely mimic the TME in ovarian cancer, we included collagen-I in the matrix. Increases in collagen-I are associated with an aging ovary, and the median age of ovarian cancer diagnosis is 63^50^. Collagen-I production is increased as ovarian cancer progresses and is associated with chemoresistance, because of this it was imperative to include collagen-I in the model^51^. Incorporating collagen-I at a low level, specifically at a 9:1 ratio of fibrin:collagen, allowed for a full microvasculature network to form (Fig. 2). The higher level of collagen-I incorporation inhibited microvasculature network formation, possibly due to compaction of collagen-I by the embedded fibroblasts which may constrict the vessels and not allow for full vascular development. The microtissue system described here represents a highly innovative in vitro model of human EOC TME that can be utilized for mechanobiological investigations of disease progression.

### Mechanical stimulation contributes to chemoresponse through HSP27

When exposed to various times of tensile strain, the SKOV-3.tr cells saw significantly more pHSP27 (Ser15) expression than the parental SKOV-3 line (Fig. 3). The SKOV-3.tr cells also saw changes in the dimer pHSP27 expression that was not observed in the SKOV-3 line. This did not translate to the total HSP27 expression where SKOV-3 cells had a higher expression for all strain exposures compared to the SKOV-3.tr and strain increased HSP27 in the SKOV-3 line. The literature shows that unphosphorylated HSP27 is associated with oligomers that can reach as high as 800 kDa; as HSP27 is phosphorylated it breaks down into lower molecular weight monomers and dimers^52^. The increase in total HSP27 expression in the SKOV-3 cell line combined with higher pHSP27 expression in the monomer and dimers for the SKOV-3.tr cell line suggests higher levels of HSP27 activity in the SKOV-3.tr cells. When the SKOV-3 and SKOV-3.tr cells were pre-treated with HSP27 inhibitor J2, HSP27 expression was only inhibited in the SKOV-3 cells that were not strained vs. strain treated cells, and this effect was not observed in the SKOV-3.tr cells. As we wanted to characterize the rapid, dynamic nature of phosphorylation of HSP27 in response to tensile strain, we did not perform any Flexcell studies for longer than 30 min. Longer strain exposures were used in microtissue studies, to ensure that EOC cell lines had fully adapted to the strain conditions prior to implantation in the microfluidic devices and mimicking conditions that generated mechanical memory in our previous studies^22^.

The ability of a cell to sense mechanical forces and retain the memory of the previous mechanical environment when placed in a new environment is called a mechanical memory. Mechanical memory works by altering the nuclear architecture of a cell, leading to epigenetic changes, and eventually changing the phenotype of the cells. These changes can be reversible or irreversible, depending on the type of mechanical cue and also the duration of the cue^53,54^ Once SKOV-3 cells were placed in the 3D TME device, they demonstrated mechanical memory represented by increased HSP27 expression in the experimental groups of cells that had been strained for 24h or 72h (Fig. 4D). This data complements our previous findings that strain treated cells can increase tumor growth and metastasis in in vivo models of ovarian cancer^22^. Furthermore, our results highlight the complexities between EOC subtypes with respect to mechanobiological responses. Namely, HSP27 and CC-3 expression levels in SKOV-3 seem to be less impacted by longer time periods of strain stimulation compared to OVCAR-8 cells, which saw a significant change in both proteins after 72 h strain (Fig. 9). Together, the data suggest that while HSP27 may play a role in mechanotransductive behaviors related to chemoresponse, these may be divergent between different subtypes of ovarian tumors. This phenonmenon needs further exploration to identify potential treatment strategies for EOC. To investigate the chemoresistance of the strained cells and determine if high HSP27 levels correlate with chemoresistance, SKOV-3 and SKOV-3.tr cells that had been strained were placed in the 3D TME model and treated with paclitaxel (Fig. 6). The combination treatment of strain and paclitaxel led to higher HSP27 levels in SKOV-3 and SKOV-3.tr experimental groups in the cells that had been stretched for 24 h compared to 72 h (Fig. 5I). HSP27 is a small protein, and it may be quickly expressed and moved to the nucleus after phosphorylation. For this reason, it would seem that for the first 24 h of strain, HSP27 is highly expressed in order to protect the cell, and after 24 h the expression of HSP27 decreases.

### Apoptosis markers are decreased in taxol-resistant cells with longer strain treatments

Cleaved caspase-3 (CC-3) was also examined as an early apoptosis marker to determine the level of cell death in the microtissues after treatment with chemotherapy. CC-3 expression was significantly higher in the SKOV-3.tr cells that were strained for 24 h compared to control (Ctl, non-strained) conditions (Fig. 6J). This suggests that the higher level of HSP27 in SKOV-3.tr cells may help protect the cells from death under longer periods of strain. While there are some differences in Ctl (non-strained) chamber levels for HSP27 (Fig. 6A,C) and CC-3 (Fig. 6E,G), this may be due to cells having more time to recover from trypsinization prior to plating on Flexcell plates (72 h vs 24 h) rather than strain treatment or cell death due to loading protocols. Furthermore, there is no difference in SKOV-3 cell viability as measured by Sytox staining for cells treated with 24 h strain vs. Ctl (non-strained) cells at 1 day or 8 days post-loading (Fig. S1). Decreased CC-3 expression in strain-treated EOCs that were subjected to paclitaxel treatment suggests that chemoresistance of the cells has increased. The 3D TME model systems recieved paclitaxel 4 days after strain treatment and device loading, representing a delay between mechanical stimulation and drug exposure. Furthermore, an additional 4 days of culture time post-paclitaxel treatment were carried out before analysis of the microtissues occurred. Therefore, it is possible that our results missed rapidly induced cell death due to drug treatment in the microtissues. As CC-3 is an early marker for apoptosis in cells and does not stain cells that may have previously gone through apoptosis, we decided to examine HSP27, CC-3, and overall cell death in cells that were subjected strain followed immediately by paclitaxel treatment (Fig. 7C). Our studies show SKOV-3.tr cells expressed lower levels of HSP27 and CC-3 after strain treatment compared to non-strained control cells. Additionally, there was slightly decreased cell death as quantified by Sytox staining in the SKOV-3.tr cells but not the parental SKOV-3 cells. While the cell death studies do not show clear chemoresistance associated with paclitaxel treatment and strain, it is important to note this experiment was a 2D study. This suggests that the 3D TME may differentially regulate mechanotransduction to drive response to treatment in EOC. These studies further support that HSP27 and CC-3 expression are regulated by mechanical strains and may play a role in chemoresistance of EOC.

### Inhibiting HSP27 alters chemoresponse in EOC cells

When the SKOV-3 cells were pre-treated with strain and J2 prior to loading in microtissues, lower the HSP27 expression was observed in the cells when they were treated with a vehicle but not when they were treated with the paclitaxel (Fig. 8). This showed that HSP27 increases in response to drug treatment even when pre-exposed to an inhibitor. The SKOV-3.tr cells did not see a change in HSP27 expression between the Veh (DMSO) and J2 treatments when treated with chemotherapy regardless of whether the cells were pre-strained or not. The SKOV-3 cells required a strain pre-treatment to disrupt the effects of the J2 when treated with paclitaxel. This shows that the drug resistant cells can more easily overcome the effects of the inhibitor than the parental line. Finally, to investigate if these aforementioned characteristics present in other histotypes of EOC, these experiments were repeated with OVCAR-8 cells which represent a serous type of ovarian cancer. After 15 min of strain, pHSP27 expression was significantly higher compared to 30 min strain treatment (Fig. 9B) with no significant differences in total HSP27 expression. After placing these cells in the 3D TME model (Fig. 9D,E), cells pre-treated with tensile strain for 72 h had significantly less CC-3 than Ctl (non-strained) cells (Fig. 9G). These same trends were observed for HSP27 expression (Fig. 9F), further supporting the role of HSP27 as a mediator of strain response in EOC. Although high levels of CC-3 were present in 72 h Ctl (non-strained) OVCAR-8 cells in TME models relative to 24 h samples (Fig. 9G), the role of strain as protective for OVCAR-8 survival is still seen when comparing between non-strained and strained cells. Furthermore, it is possible that the OVCAR-8 cells are more sensitive or less robust than SKOV-3 with respect to long-term culture in the 3D TME models. We do not believe this is a limitation in the microtissue platform due to nutrient diffusion issues, as there is little cell death observed in our control viability studies which indicates all regions of the microtissues are receiving media (Fig. S1). Overall, our results may point to a protective mechanism that prolonged tensile strain has on OVCAR-8 cells with decreases in markers of stress, early apoptosis, and cell death present in cells treated with tensile strain. While SKOV-3 and OVCAR-8 cells showed different results in relation to HSP27 expression and phosphorylation patterns, these cell lines represent two different histological subtypes of ovarian cancer that are treated as distinct diseases. Therefore, the differing results are not surprising and may help differentiate therapeutic strategies targeting HSP27 between different subtypes of EOC.

Taken together, this study provides evidence that HSP27 may have a protective role in both serous and non-serous EOC cells regulated by tensile forces. Phosphorylated HSP27 expression is higher in SKOV-3 cells that are exposed to tensile stress than the chemoresistant cell line SKOV-3.tr. Total HSP27 expression is higher in SKOV-3.tr cells exposed to tensile stress than SKOV-3 cells, possibly because the HSP27 has already been phosphorylated and moved to the nucleus and subsequently degraded^52,55^. These early experiments with increased HSP27 expression were confirmed in a 3D TME model, with samples showing a decrease in the early apoptosis marker CC-3, further supporting a potential role of HSP27 in EOC chemoresistance to paclitaxel. Our results show alterations in HSP27 expression and activation, linking these changes to regulation of EOC response to paclitaxel treatment in 3D models. The short term studies of phosphorylation dynamics of HSP27, with and without strain as well as when inhibited with J2, highlight the dynamic nature of the mechanoregulation of this factor within cells. Limitations in the Flexcell system, namely difficulty in imaging and lack of 3D matrix cues, prompted us to further explore EOC behvaiors and response to treatment in a 3D microtissue co-culture model that better mimics the ovarian TME. While some differences in timing for treatments, namely length of strain and length of J2 treatment, exist between 2D and 3D experimental setups, the data provide a robust understanding of the interaction of mechanical strain with HSP27 regulation and response to chemotherapeutic treatment.

This study aimed to elucidate the effects of tensile strain on HSP27 levels and the relationship to chemoresistance. Using SKOV-3, SKOV-3.tr, and OVCAR8 cell lines representing two different histological subtypes of ovarian cancer, as well as a chemoresistant subtype, our studies demonstrated the role of biomechanical stimulation on HSP27 expression in a 3D TME model. Through protein analysis and experiments using a 3D TME microfluidic model, the expression of HSP27 in cells placed under tensile strains was explored. It was found that phosphorylated HSP27 expression was higher in SKOV-3 cells and total HSP27 expression was higher in SKOV-3.tr cells. In the 3D TME microfluidic model, cells that were placed under tensile strain for a shorter time period had higher expression of HSP27 but also higher apoptosis, whereas cells placed under tensile strain for longer time periods had lower expression of HSP27 and lower apoptosis. This may be due to a protective mechanism of HSP27 that extends past its expression. Further, this may suggest a stabilization period after imposition of tensile strains where HSP27 decreases but the chemoresistant mechanisms remain. This may be why SKOV-3.tr cells demonstrate paclitaxel resistance and lower HSP27 levels after strain and paclitaxel treatments in the 3D microtissue models. These findings provide insight into a mechanical signaling link between HSP27 expression and chemoresistance of EOC. Future directions for this work involve validating the mechanism of mechanically-induced HSP27 expression and its relationship to chemoresistance in mouse models of ovarian cancer progression. However, as mouse models do not provide full control over biomechanical features in the same way our 3D microtissue model does, the results presented here are crucial for development of appropriate in vivo experiments to truly understand EOC biomechanical regulation. Ultimately, this work lays the foundation for exploration of novel targeting strategies designed to combat EOC chemoresistance and recurrence by targeting the mechanobiological factors that drive disease progression.

## Methods

### Cell culture

The SKOV-3 ovarian cancer cell line was provided by Dr. Michael Birrer (University of Arkansas); the taxol-resistant SKOV-3.tr ovarian cancer cell line was provided by Dr. Rebecca Arend (University of Alabama at Birmingham); and the OVCAR-8 cell line was provided by Dr. Geeta Mehta (University of Michigan). Human microvascular endothelial cells (HMECs, ATCC CRL-3243) and normal human lung fibroblasts (NHLFs, Lonza, CC-2512 Lot #:19TL148581) were used to create a 3D vasculature in the microfluidic devices based on a previously designed microtissue model of the TME^49^. SKOV-3, SKOV-3.tr, and HMECs are immortalized cell lines; NHLFs were used between passages 4–8. SKOV-3 and OVCAR-8 cells were cultured with RPMI 1640 1X media with a supplement of 10% FBS and 1% penicillin/streptomycin. SKOV-3.tr cells were also cultured with RPMI 1X medium with a supplement of 10% FBS and 1% penicillin/streptomycin with an addition of 150 ng/mL of paclitaxel. HMECs were cultured with MCDB 131 supplemented with 0.002% EGF, 1% Hydrocortisone, 10% heat-inactivated FBS, and 5% L-Glutamine. NHLFs were cultured with DMEM high glucose supplemented with 10% non-heat inactivated FBS, 2% sodium pyruvate, 1% L-glutamine, 1% penicillin/streptomycin, 1% non-essential amino acids. All cell types were incubated at 37°C and 5% CO_2_. For all studies where paclitaxel was used as a treatment, a concentration of 1 μM was utilized for 24 h.

### Tensile strain system

The commercially-available Flexcell 6000-T system was used to apply an oscillatory tensile force to a confluent monolayer of ovarian cancer cells. For all experiments, 5 × 10^5^ cells were seeded in each well of a collagen-I coated uniaxial Flexcell plate (UF-4001C) and placed in the incubator (37°C, 5% CO_2_) for 24 h to allow the cells to grow to ~ 80% confluence. The plates were then placed on the baseplate in an incubator (37°C, 5% CO_2_) for straining. All cells were treated with oscillatory stretch with a magnitude of 10% elongation and frequency of 0.3 Hz. These parameters mimic cell-induced strains previously studied^8^ and the rate of normal human respiration. This rate of strain was chosen to mimic a physiologically-relevant frequency for the abdominal cavity and because the lungs are a site of metastasis for ovarian cancer^56^. Flexcell experiments ran for either 5, 15, or 30 min for Western blot analysis or 24 h or 72 h for 3D microfluidic device experiments. Cells for control samples were seeded on the same flexible membrane plates and placed in the same incubator as the experimental (strained) plates for the same periods of time but were not subjected to tensile strain.

### Western blot analysis

Western Blot analysis was performed to determine levels of phosphorylated HSP27 (Ser15) and total HSP27 in samples of SKOV-3, SKOV-3.tr and OVCAR-8 cells stimulated with strain for 5-30 min. To test inhibition of HSP27, we used a small molecule inhibitor, J2, prior to Western blot analysis of phosphorylated and total HSP27 (MedChemExpress, HY-124653) In the inhibitor studies, cells were seeded onto the FlexCell plates and allowed to adhere for 4 h prior to 10 μM J2 treatment or vehicle control (Veh, DMSO). Note: For our studies, Veh = DMSO used as a control for J2; VEH = DMSO vehicle used as a control for paclitaxel treatment. Cells were treated for 24 h prior to strain treatment, followed by lysing. Cell lysates were generated using 1X RIPA buffer with protease and phosphatase inhibitors (HALT Solution, ThermoFisher 78440), and standard protocols were used to process the samples. Briefly, lysates were run on 12 or 15% SDS-PAGE gels before transfer to a PDVF membranes. For phosphorylation studies, 1% BSA in TBST was used as the blocking and antibody dilution solution. Primary antibodies used were rabbit anti-HSP27 (phospho-S15) antibody (Abcam ab76313, RRID: AB_1523792) at a 1:500 dilution, rabbit anti-HSP27 antibody (Abcam ab109376, RRID: AB_10865046) at 1:1000 dilution, and mouse anti-β-Actin antibody (Sigma Aldrich A1978, RRID: AB_476692) as a loading control at a 1:40,000 dilution. All HRP-linked secondary antibodies were used at 1:2000 (Anti-Rabbit, Cell Signaling Technology 7074S, RRID: AB_2099233 or Anti-Mouse, Cell Signaling Technology 7076S, RRID: AB_330924). For phosphorylation studies, Super Signal Femto Substrate was used (FisherSci PI34095) After phosphorylation analysis, membranes were stripped with Re-Blot solution (Sigma-Aldrich 2504). Total proteins were measured via Pierce ECL solution (FisherSci PI32106). Densitometry was performed using FIJI.

### 3D TME model

Design and manufacturing of the 3D microfluidic device used in these experiments has been detailed previously^49^; this system is designed with three microtissue chambers in series and permits high levels of control over matrix components, cell loading and interstitial flow (Fig. 1). Briefly, polydimethyl siloxane (PDMS, Sylgard 184, Dow Corning) was cast with a base-to-curing agent ratio of 10:1 on a silicon wafer mold previously fabricated using soft lithography and cured at 65°C for at least 3 h. PDMS devices were then plasma bonded to glass slides. To prepare devices for cell and gel loading, cells were trypsinzed and resuspended at appropriate concentrations in pre-gel solution containing fibrinogen (Sigma-Aldrich, F8630) and collagen-I (Corning, Ref: 35,429). The pre-gel suspension was mixed with thrombin (Sigma-Alrich, T4648) diluted in DPBS + 0.1% BSA such that a final concentration of 3 U/mL thrombin was achieved. The cell-gel solution was quickly injected with a micropipette into a loading port in the PDMS^57^. Center chambers of devices were loaded with a 9:1 fibrin:collagen-I ratio (10 mg/mL total ECM protein) and a final concentration of 1 × 10^7^ cells/mL for a 1:1 ratio of HMECs and NHLFs (Fig. 1A). NHLFs are added to act as stromal cells, to support self-assembly of HMECs into blood vessels and provide stability to the vasculature over the course of several days^58,59^; additionally, since the lungs are the site of ovarian metastatic growth, the TME model represents a physiologically-relevant stromal component^56^. Side chambers were loaded with the same matrix and 5 × 10^3^ cancer cells that had either been subjected to tensile strain via the Flexcell system for 24 h or 72 h or had not been subjected to strain (Ctl) (Fig. 1A). To inhibit HSP27 in the devices, we utilized the J2 inhibitor at 10 μM. For these studies, cells were treated with J2 or Veh (DMSO) for 24 h prior to strain which was maintained for the duration of strain treatment prior to loading (Fig. 8). The devices in the inhibitor study received 10 μM J2 treatment or Veh (DMSO) for days 1–3, then paclitaxel or VEH (DMSO) treatment on day 4, and EGM-2 only on days 5–8. Devices were fed daily with fresh EGM-2 media (Lonza, CC-3162), supplemented as noted above, for 8 days with flow setup so that media moved from the center chamber into the side chambers only, effectively limiting diffusion of any secreted factors from the cancer cells from entering the center chamber (Fig. 1B). This “outward” flow regime also generates equal magnitude interstitial flows into the side chambers, creating a built-in control where shear stresses experienced in one chamber are identical in magnitude, though opposite in direction, to the other side chamber; therefore, flow is controlled for as a biological variable within our system. Moreover, the flow setup provides a physiologically-normal range (0–0.2 μm/s) for interstitial flow rates as previously described^57^. Devices were then fixed and stained for immunofluorescence imaging.

To quantify viability in 3D TME models after strain treatments, we utilized a Sytox (Invitrogen S11381) staining protocol. In this experiment, the cells were strained for 24 h , or were cultured on Flexcell plates but received no strain (Ctl) before being harvested for loading into microfluidic devices. The cells were loaded into the center chamber as described before with 9:1 fibrin:collagen-I ratio and 5X10^3^ cells. The side chambers were loaded with only the matrix and no cells. The devices were fed with RPMI. Devices were treated with Sytox (0.5 μM) and Hoescht (1 μM) staining for 3 h prior to image analysis as described before. Cell death was normalized to total cell count in the center chamber. Samples were stained and imaged on either day 1 or day 8 post-loading.

### Staining and immunofluorescence imaging

Immunofluorescence protocols for the 3D microtissue system were previously described^49^. Briefly, all reagents given to the devices described herein were administered for 48 h and incubated at 4°C. To prepare the devices for antibody staining, the devices were first fixed with 10% formalin, then permeabilized with PBS + 0.5% Tween-20 to allow for intracellular staining, blocked with 2% BSA in PBS + 0.1% Tween-20, before addition of primary antibodies, washed with PBS + 0.1% Tween-20, and followed by secondary antibodies. After secondary antibodies were removed, a PBS + 0.1% Tween-20 wash containing 1:1000 DAPI was added to devices and allowed to sit for at least 1 h prior to imaging. Antibodies used in these studies include the following: mouse anti-HSP27 (Invitrogen MA3-015, RRID: 325,463) at a 1:500 dilution, rabbit anti-Cleaved Caspase-3 (Abcam ab32042, RRID: 725,947) at a 1:250 dilution, CD31 (Fisher Scientific, PIMA513188) at a 1:200 dilution, Alexa Fluor 488 goat anti-rabbit (ThermoFisher A11029) or Alexa Fluor 555 donkey anti-mouse IgG (ThermoFisher A31570) both at a 1:500 dilution. An Olympus IX83 inverted epifluorescent microscope was used for imaging and FIJI was used to process and stitch the images^60^. For all microtissue devices, a 100 μm z-stack was collected with step size 2 μm.

### 2D cell death analysis

To determine if differences in early apoptosis and cell death markers could be seen, we completed 2D studies of paclitaxel on SKOV-3 and SKOV-3.tr cells. Cells were plated onto Flexcell plates as previously describe and exposed to 10% strain at 0.3Hz for 24h. Then, the cells were harvested via trypsinization and seeded at 1 × 10^5^ cells/well in a 24 well plate. The cells were allowed to adhere to the plate for 6 h before treatment with 1 μM paclitaxel or vehicle control. After 24 h of treatment, the cells were washed with PBS, fixed for 20min using 10% buffered formalin, permeabilized for 20 min using 0.5% Tween-20 in PBS, blocked using 2% BSA in 0.1% Tween-20 in PBS. Cells were incubated in primary antibody (HSP27, 1:500 Inivtrogen MA3-015; cleaved caspase-3, 1:250, ab32042) for 20min at room temperature. Secondary antibody incubation was 20 min at room temperature (1:500 Alexa Fluor 488 goat anti-rabbit, Invitrogen A11034 and 1:500 Alexa Fluor 555 donkey anti-mouse, Invitrogen A31570). Cell nuclei were counterstained with DAPI. A second series of studies used Sytox (Invitrogen, 50-113-7614) and Hoescht 33342 (Thermo Scientific, PI62249 at 1 μM) for 30 min to measure cell death in response to paclitaxel or vehicle controls.

### ImageJ and AngioTool analysis

In FIJI, image stacks were stitched together to create full-length representations of each device and then were processed using the Max Intensity Z-Projection function to create a 2D image for each microtissue^61^. Background subtraction was uniformly applied to each 2D projection within experiment sets, and images were thresholded to create binary masks. Individual chambers were analyzed with the Analyze Particles feature to quantify relative levels of HSP27 and Cleaved Caspase-3 (CC-3) in side chambers of devices and normalized to nuclear counts within the chamber. Thus, each device acted as its own control, with one side chamber was loaded with cells that had been strained while the other side chamber received unstrained, control cells. AngioTool was used to map skeletonization of blood vessels and to determine blood vessel growth and mean lacunarity^40^. For the Sytox analysis, the number of Sytox-postive cell nuclei were normalized to total nuclei count for each image.

### Statistical analysis

All results are presented as averages plus or minus the standard error of the mean (SEM) for all sample types. A minimum of 3 samples were used in each study. Data was checked for normality using a Shapiro–Wilk test. If all sample groups within an experiment were normal, ANOVA with post-hoc Tukey HSD tests were performed. If any samples were non-normally distrubited, data was analyzed via Kruskal–Wallis testing followed by Dunn’s tests. For samples comparing only two conditions, either a student’s t-test assuming unequal variances or a Mann–Whitney paired test were used. Specific statistical tests for reach study are shown in the figure captions.

### Supplementary Information

Supplementary Information.

## Data Availability

Raw images and data sets generated by these studies are available upon reasonable request to the senior author.

## Abbreviations

CC-3: Cleaved caspase-3
EOC: Epithelial Ovarian Cancer
HSP27: Heat Shock Protein 27
TME: Tumor microenvironment

## References

[1] Sung H (2021). Global Cancer Statistics 2020: GLOBOCAN estimates of incidence and mortality worldwide for 36 cancers in 185 countries. CA Cancer J. Clin..

[2] Agarwal R, Kaye SB (2003). Ovarian cancer: Strategies for overcoming resistance to chemotherapy. Nat. Rev. Cancer.

[3] Ozols RF (2023). Phase III trial of carboplatin and paclitaxel compared with cisplatin and paclitaxel in patients with optimally resected stage III ovarian cancer: A gynecologic oncology group study. J. Clin. Oncol..

[4] Abal M, Andreu JM, Barasoain I (2003). Taxanes: Microtubule and centrosome targets, and cell cycle dependent mechanisms of action. Curr. Cancer Drug Targets.

[5] Tse JM (2012). Mechanical compression drives cancer cells toward invasive phenotype. Proc. Natl. Acad. Sci. USA.

[6] Samuel MS (2011). Actomyosin-mediated cellular tension drives increased tissue stiffness and beta-catenin activation to induce epidermal hyperplasia and tumor growth. Cancer Cell.

[7] Jain RK (1987). Transport of molecules across tumor vasculature. Cancer Metastasis Rev..

[8] Sewell-Loftin MK (2017). Cancer-associated fibroblasts support vascular growth through mechanical force. Sci. Rep..

[9] McKenzie AJ (2018). The mechanical microenvironment regulates ovarian cancer cell morphology, migration, and spheroid disaggregation. Sci. Rep..

[10] le Noble F (2004). Flow regulates arterial-venous differentiation in the chick embryo yolk sac. Development.

[11] Engler AJ (2006). Matrix elasticity directs stem cell lineage specification. Cell.

[12] Jaalouk DE, Lammerding J (2009). Mechanotransduction gone awry. Nat. Rev. Mol. Cell Biol..

[13] Iskratsch T, Wolfenson H, Sheetz MP (2014). Appreciating force and shape-the rise of mechanotransduction in cell biology. Nat. Rev. Mol. Cell Biol..

[14] Montagner, M. and S. Dupont, *Mechanical forces as determinants of disseminated metastatic cell fate.* Cells, 2020. **9**(1). 10.3390/cells9010250 PMC7016729 31963820

[15] Lehman HL (2013). Modeling and characterization of inflammatory breast cancer emboli grown in vitro. Int. J. Cancer.

[16] Ren J (2015). An atomic force microscope study revealed two mechanisms in the effect of anticancer drugs on rate-dependent young's modulus of human prostate cancer cells. PLoS One.

[17] Wang, Y., et al., *Mechanical strain induces phenotypic changes in breast cancer cells and promotes immunosuppression in the tumor microenvironment.* Lab Invest, 2020. 10.1038/s41374-020-0452-1 PMC7686122 32572176

[18] Chen YS (2019). Locally targeting the IL-17/IL-17RA axis reduced tumor growth in a murine B16F10 melanoma model. Hum Gene Ther..

[19] Hendricks P (2012). Effects of respiratory mechanical forces on the pharmacological response of lung cancer cells to chemotherapeutic agents. Fundam. Clin. Pharmacol..

[20] Ip CK (2016). Stemness and chemoresistance in epithelial ovarian carcinoma cells under shear stress. Sci. Rep..

[21] Novak, C.M., et al., *Compressive Stimulation Enhances Ovarian Cancer Proliferation, Invasion, Chemoresistance, and Mechanotransduction via CDC42 in a 3D Bioreactor.* Cancers (Basel), 2020. **12**(6). 10.3390/cancers12061521 PMC7352213 32532057

[22] Martinez, A., et al., *Understanding the effect of mechanical forces on ovarian cancer progression.* Gynecol Oncol, 2021. 10.1016/j.ygyno.2021.04.003 PMC9115803 33888338

[23] Avraham-Chakim L (2013). Fluid-flow induced wall shear stress and epithelial ovarian cancer peritoneal spreading. PLoS One.

[24] Masiello, T., et al., *A dynamic culture method to produce ovarian cancer spheroids under physiologically-relevant shear stress.* Cells, 2018. **7**(12). 10.3390/cells7120277 PMC6316168 30572633

[25] Carduner L (2014). Ascites-induced shift along epithelial-mesenchymal spectrum in ovarian cancer cells: enhancement of their invasive behavior partly dependant on alphav integrins. Clin. Exp. Metastasis.

[26] Rizvi I (2013). Flow induces epithelial-mesenchymal transition, cellular heterogeneity and biomarker modulation in 3D ovarian cancer nodules. Proc. Natl. Acad. Sci. USA.

[27] Garrido C (2002). Size matters: Of the small HSP27 and its large oligomers. Cell Death Differ..

[28] Rogalla T (1999). Regulation of Hsp27 oligomerization, chaperone function, and protective activity against oxidative stress/tumor necrosis factor alpha by phosphorylation. J. Biol. Chem..

[29] Lianos GD (2015). The role of heat shock proteins in cancer. Cancer Lett..

[30] Zhang, B., et al., Heat shock protein 27 phosphorylation regulates tumor cell migration under shear stress. Biomolecules, 2019. **9**(2). 10.3390/biom9020050 PMC6406706 30704117

[31] Kim KK, Kim R, Kim SH (1998). Crystal structure of a small heat-shock protein. Nature.

[32] Brunet Simioni M (2009). Heat shock protein 27 is involved in SUMO-2/3 modification of heat shock factor 1 and thereby modulates the transcription factor activity. Oncogene.

[33] Carver JA (1995). On the interaction of alpha-crystallin with unfolded proteins. Biochim. Biophys. Acta.

[34] MacRae TH (2000). Structure and function of small heat shock/alpha-crystallin proteins: Established concepts and emerging ideas. Cell Mol. Life Sci..

[35] Langdon SP (1995). Expression of the heat shock protein HSP27 in human ovarian cancer. Clin. Cancer Res..

[36] Song TF (2009). Small interfering RNA-mediated silencing of heat shock protein 27 (HSP27) Increases chemosensitivity to paclitaxel by increasing production of reactive oxygen species in human ovarian cancer cells (HO8910). J. Int. Med. Res..

[37] Lu H (2016). HSP27 knockdown increases cytoplasmic p21 and cisplatin sensitivity in ovarian carcinoma cells. Oncol. Res..

[38] Williams RM (2010). Strategies for high-resolution imaging of epithelial ovarian cancer by laparoscopic nonlinear microscopy. Transl. Oncol..

[39] Nadiarnykh O (2010). Alterations of the extracellular matrix in ovarian cancer studied by Second Harmonic Generation imaging microscopy. BMC Cancer.

[40] Zudaire E (2011). A computational tool for quantitative analysis of vascular networks. PloS one.

[41] Novak, C.M., et al., Compressive stimulation enhances ovarian cancer proliferation, invasion, chemoresistance, and mechanotransduction via CDC42 in a 3D bioreactor. Cancers 2020. **12**(6). 10.3390/cancers12061521 PMC7352213 32532057

[42] Yang J (2004). Twist, a master regulator of morphogenesis, plays an essential role in tumor metastasis. Cell.

[43] Farge E (2003). Mechanical induction of twist in the drosophila foregut/stomodeal primordium. Curr. Biol..

[44] Hoffman L (2017). Mechanical signals activate p38 MAPK pathway-dependent reinforcement of actin via mechanosensitive HspB1. Mol. Biol. Cell.

[45] Calvo F (2013). Mechanotransduction and YAP-dependent matrix remodelling is required for the generation and maintenance of cancer-associated fibroblasts. Nat. Cell Biol..

[46] Chang YC (2019). Hippo signaling-mediated mechanotransduction in cell movement and cancer metastasis. Front. Mol. Biosci..

[47] Rogalla T (1999). Regulation of Hsp27 oligomerization, chaperone function, and protective activity against oxidative stress/tumor necrosis factor α by phosphorylation. J. Biol. Chem..

[48] Arrigo A-P, Préville X (1999). Role of Hsp27 and related proteins. Stress proteins.

[49] Sewell-Loftin MK (2020). Micro-strains in the extracellular matrix induce angiogenesis. Lab. Chip..

[50] Siegel RL, Miller KD, Jemal A (2016). Cancer statistics, 2016. CA Cancer J. Clin..

[51] Kramer M, Criswell A, Sewell-Loftin MK (2023). Biomaterial considerations for ovarian cancer models. Front. Mater..

[52] Katsogiannou M, Andrieu C, Rocchi P (2014). Heat shock protein 27 phosphorylation state is associated with cancer progression. Front Genet.

[53] Nasrollahi S (2017). Past matrix stiffness primes epithelial cells and regulates their future collective migration through a mechanical memory. Biomaterials.

[54] Scott AK, Rafuse M, Neu CP (2023). Mechanically induced alterations in chromatin architecture guide the balance between cell plasticity and mechanical memory. Front. Cell Dev. Biol..

[55] Parcellier A (2003). HSP27 is a ubiquitin-binding protein involved in I-kappaBalpha proteasomal degradation. Mol. Cell Biol..

[56] Deng K (2018). Sites of distant metastases and overall survival in ovarian cancer: a study of 1481 patients. Gynecol. Oncol..

[57] Sewell-Loftin, M.K., et al., Micro-strains in the extracellular matrix induce angiogenesis. Lab Chip. 2020. 10.1039/d0lc00145g PMC7659465 32614340

[58] Moya ML, Alonzo LF, George SC (2014). Microfluidic device to culture 3D in vitro human capillary networks. Methods Mol. Biol..

[59] Moya ML (2013). In vitro perfused human capillary networks. Tissue Eng. C Methods.

[60] Preibisch S, Saalfeld S, Tomancak P (2009). Globally optimal stitching of tiled 3D microscopic image acquisitions. Bioinformatics (Oxford, England).

[61] Preibisch S, Saalfeld S, Tomancak P (2009). Globally optimal stitching of tiled 3D microscopic image acquisitions. Bioinformatics.

