# Supplementary material for: Mechanical activation and expression of HSP27 in epithelial ovarian cancer
Source: Sci Rep. 2024 Feb 3;14:2856. doi: 10.1038/s41598-024-52992-7 (PMC10838328; doi:10.1038/s41598-024-52992-7)
Supplement: Supplementary file 1 — Supplementary Information. [file 41598_2024_52992_MOESM1_ESM.pdf]

Supplemental Information: Mechanical activation and expression of HSP27 in epithelial ovarian cancer

Molly Buckley[+1], Maranda Kramer[+1], Bronte Johnson[1], Gillian Huskin[1], Joel Berry[1,2], and Mary Kathryn Sewell-Loftin*[1,2]

+ Authors Contributed Equally to the Manuscript

[1]Department of Biomedical Engineering, University of Alabama at Birmingham, Birmingham, AL 35294; [2]O'Neal Comprehensive Cancer Center, University of Alabama at Birmingham, Birmingham AL 35233

*Corresponding Author:
Mary Kathryn Sewell-Loftin
Assistant Professor
Department of Biomedical Engineering
University of Alabama at Birmingham
1824 6th Avenue South
Wallace Tumor Institute, Room 630A
Birmingham, AL 35294
Telephone: (205) 934-0198
Email: mksewellloftin@uab.edu
ORCID: 0000-0003-1985-6899

Supplemental Figures.

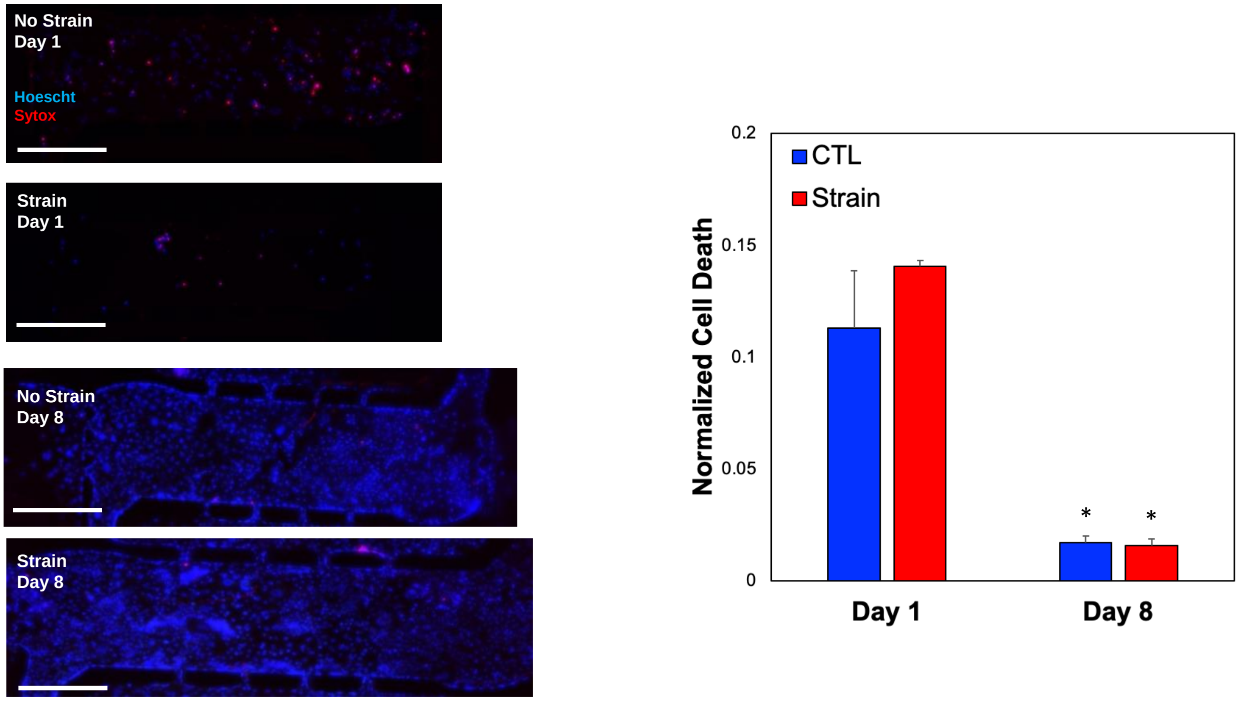

Figure S1. Viability of SKOV-3 cells in microfluidic devices after strain. As a control experiment, SKOV-3 cells were loaded into the center chamber as previous described after 24hr of strain treatment. The samples were stained with Sytox and Hoescht for 3 h to quantify cell death. (Left) Representative fluorescent images of Sytox (red) and Hoescht (blue). Scale bar = 200μm. (Right) Quantification of cell death normalized to total cell count. Data shown as average + SEM, N=3; *p<0.05. A Student's t-test assuming unequal variances was used to compare groups.

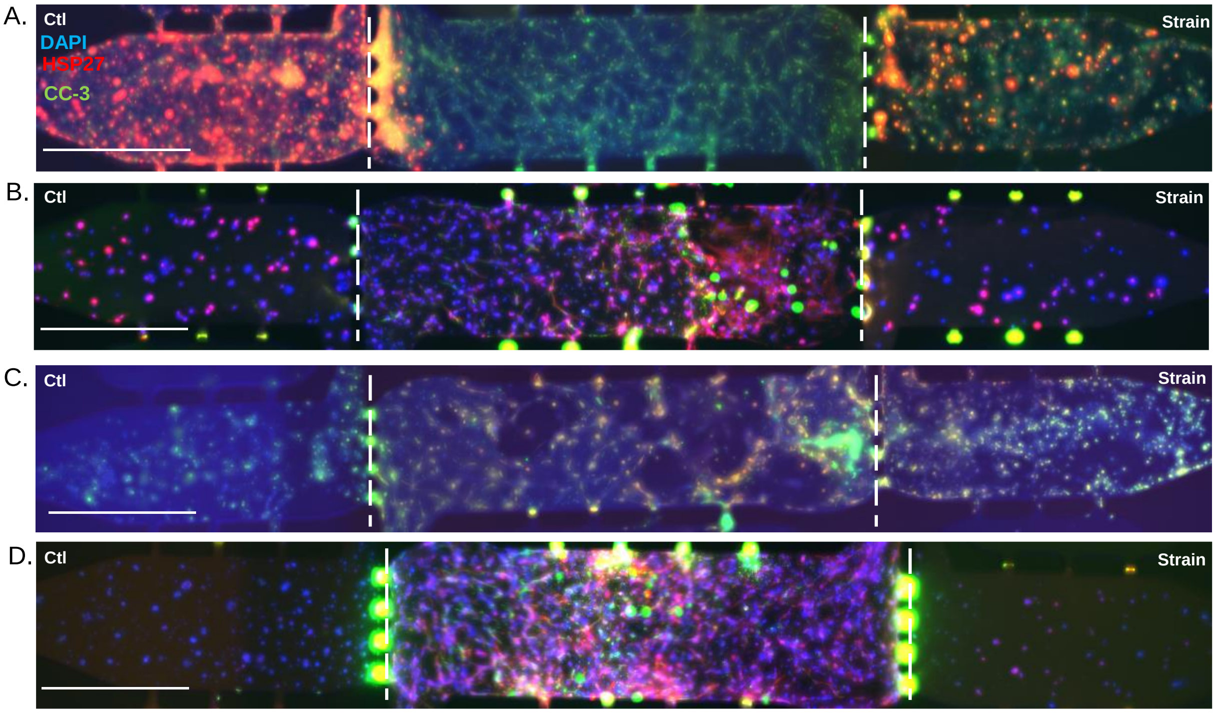

Figure S2. Representative full-length fluorescence images of 3D microtissues models. Systems are loaded with either SKOV-3 cells or SKOV-3.tr cells in side chambers, with NHLFs and HMECs in center chamber before treatment with paclitaxel. Systems are stained for HSP27 (TXRED), CC-3 (GFP), and DAPI. (A,B) Devices with SKOV-3 cells placed under oscillatory tensile strain for 24 h (A) or 72 h (B) vs control (non-strained cells) in the side chambers. (C,D) Devices with SKOV-3.tr cells placed under oscillatory tensile strain for 24 h (C) or 72 h (D) vs control (non-strained cells) in the side chambers. Scale bar = 200 µm. White dashed lines show interfaces between chamber regions. These images contain all chambers and all fluorescence channels from Fig. 6.

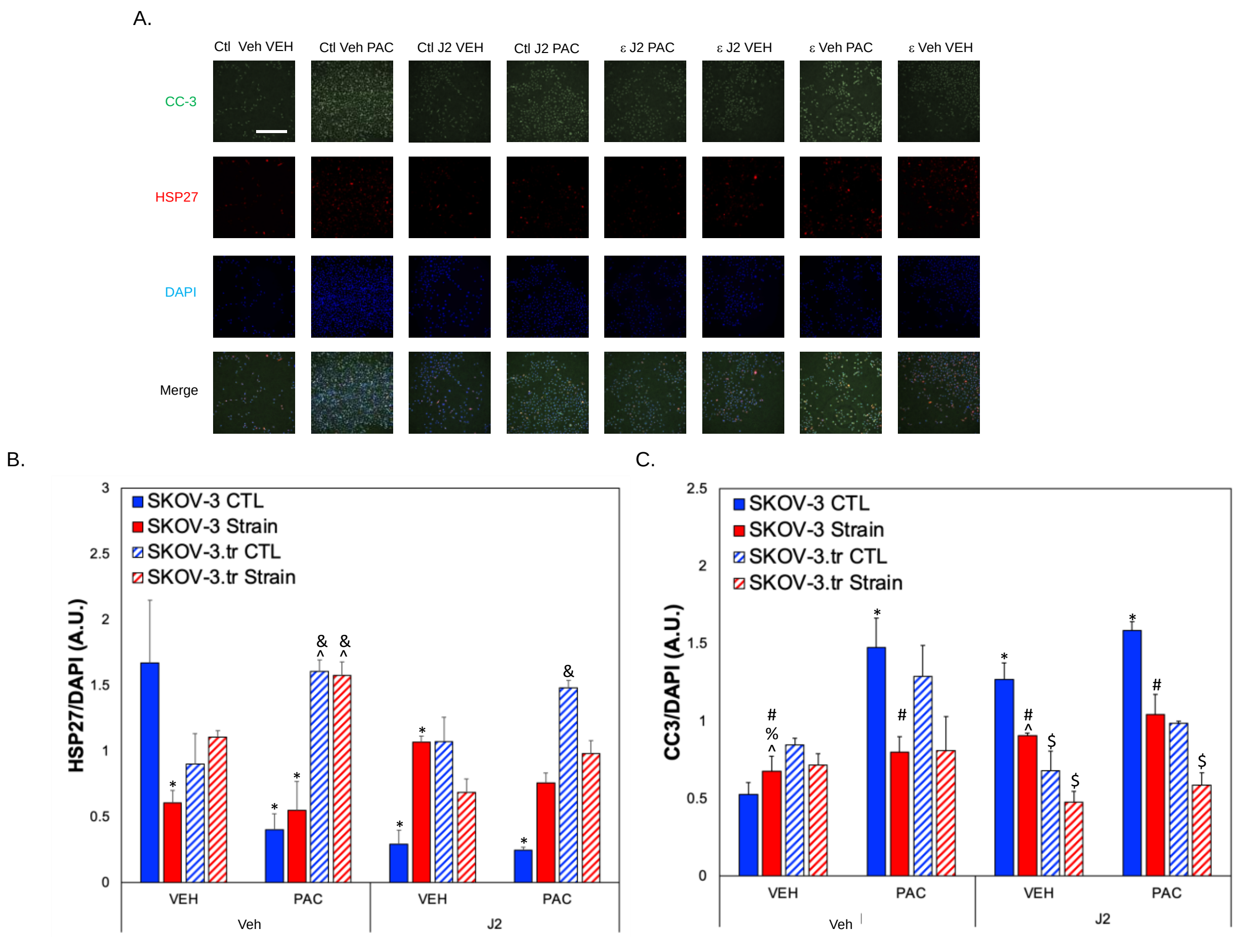

Figure S3. **Quantification of HSP27 and CC-3 in 2D Studies.** (A) Representative 2D images of SKOV-3 cells stained for HSP27, CC3, and DAPI. Ctl = No Strain; ε = strain treatment; Veh = Vehicle control for J2 (DMSO); J2 = HSP27 inhibitor at 10μM for 24h; VEH = Vehicle control for Paclitaxel (DMSO); PAC = 1 μM paclitaxel for 24h. Scale bar = 500 μm. (B) Quantification of HSP27 from (A). ^ p<0.05 compared to SKOV-3.tr CTL Veh-VEH; & p<0.05 compared to SKOV-3.tr Strain J2 VEH ; * p<0.05 compared to SKOV-3 CTL Veh-VEH; Samples were compared with ANOVA followed by post-hoc Tukey HSD tests. (C) Quantification of CC-3 from (A). * p<0.05 compared to SKOV-3 CTLVeh-VEH; ^ p<0.05 compared to SKOV-3 CTL Veh-PAC; % p<0.05 compared to SKOV-3 CTL J2 VEH; # p<0.05

compared to SKOV-3 CTL J2 PAC; \$ $p<0.05$ compared to SKOV-3.tr CTL Veh-PAC. Samples were compared with ANOVA followed by post-hoc Tukey HSD tests. All data shown as average + SEM, N=3 samples.

Unmodified:

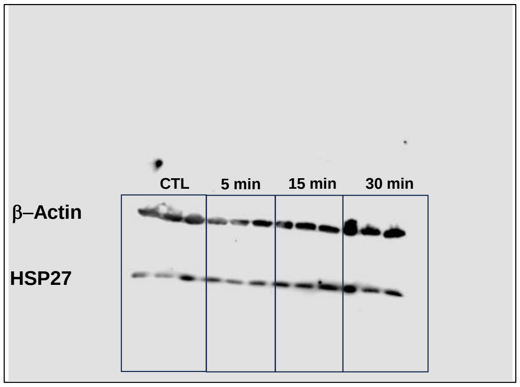

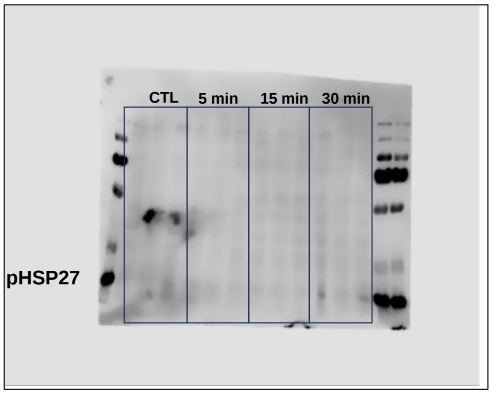

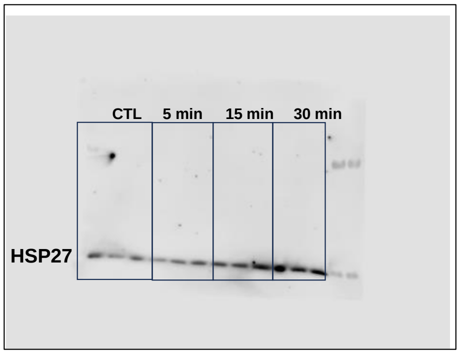

Contrast Enhanced:

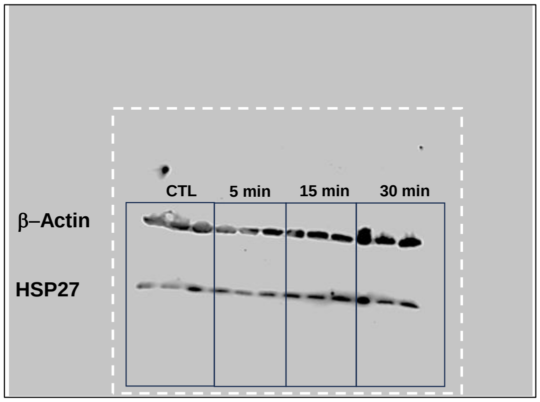

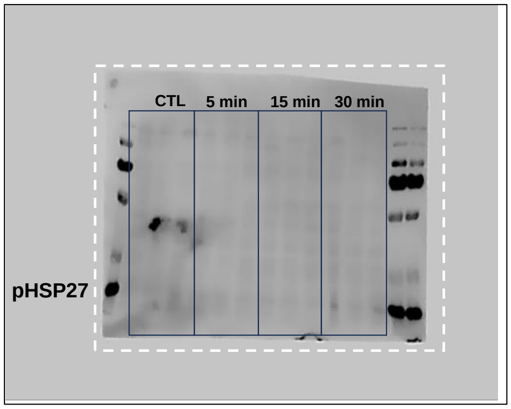

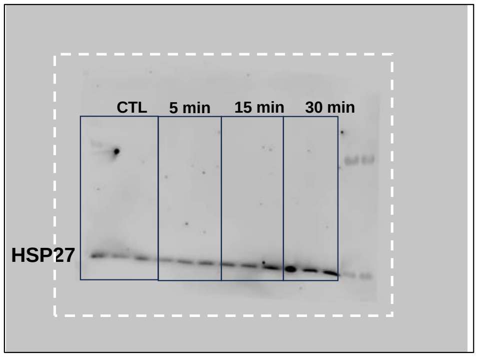

Figure S4. Uncropped Western blot images for SKOV-3 cells treated with strain. Data shown in Fig. 3A, including both pHSP27, total HSP27, and β-actin loading controls. All samples originated in the same experiment, and membranes were processed in parallel. Additional bands are internal controls or different experiments not included in this manuscript. Different exposure times are shown for the different proteins, due to inconsistencies with binding affinities and resulting intensities for the different primary antibodies. Total HSP27 was measured on the same membrane after processing with stripping solution; this is the reason why the image is not perfectly aligned with the β-actin and pHSP27 images. Dark bands on sides are ladder (PageRuler Plus, ThermoFisher #26619). All images have been post-processed with the same techniques to ensure accurate quantification and comparisons. For clarity and completeness, the top three images are fully uncropped and unmodified images from the Licor software, annotated to show bands of interest. The auto contrast on this system makes it difficult to see the edges of membranes in some cases, so we have provided contrast enhanced images (bottom 3 figures) with white dashed outlines to indicate membrane edges.

Unmodified:

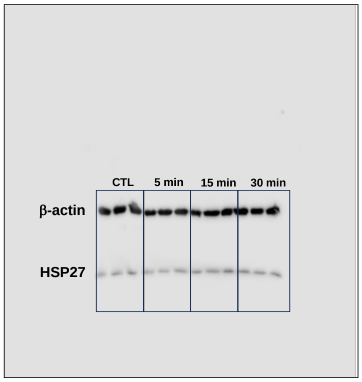

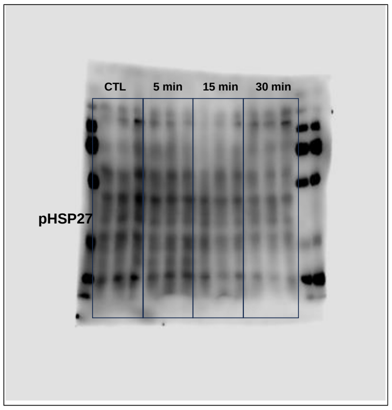

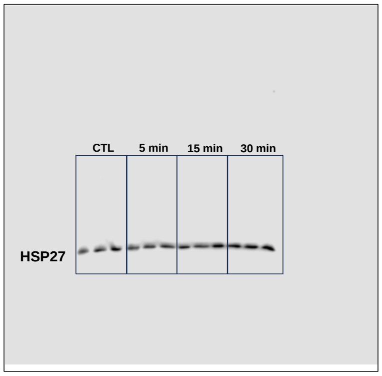

Contrast Enhanced:

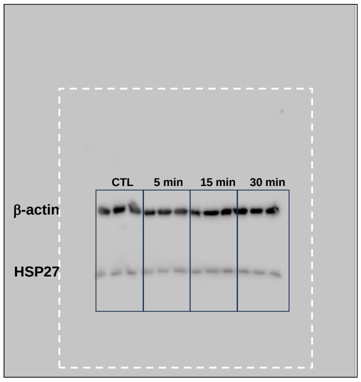

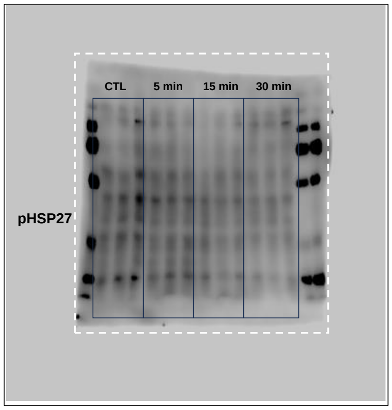

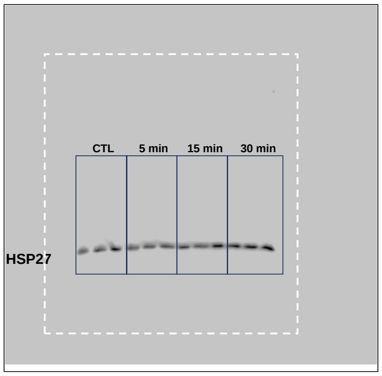

Figure S5. Uncropped Western blot images for SKOV-3.tr cells treated with strain. Data shown in Fig. 3B, including both pHSP27, total HSP27, and β-actin loading controls. All samples originated in the same experiment, and membranes were processed in parallel. Different exposure times are shown for the different proteins, due to inconsistencies with binding affinities for the different primary antibodies. Additional bands are internal controls or different experiments not included in this manuscript. Total HSP27 was measured on the same membrane after processing with stripping solution; this is the reason why the image is not perfectly aligned with the β-actin and pHSP27 images. All images have been post-processed with the same techniques to ensure accurate quantification and comparisons. For clarity and completeness, the top three images are fully uncropped and unmodified images from the Licor software, annotated to show bands of interest. The auto contrast on this system makes it difficult to see the edges of membranes in some cases, so we have provided contrast enhanced images (bottom three figures) with white dashed outlines to indicate membrane edges.

Unmodified:

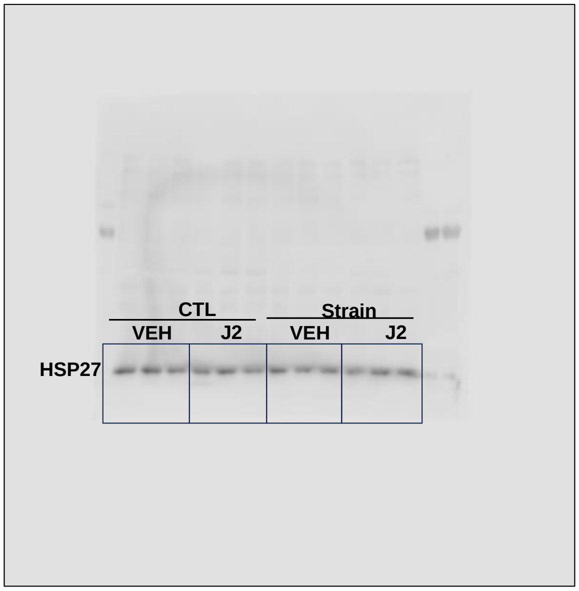

CTL
Strain
VEH
J2
VEH
J2
HSP27

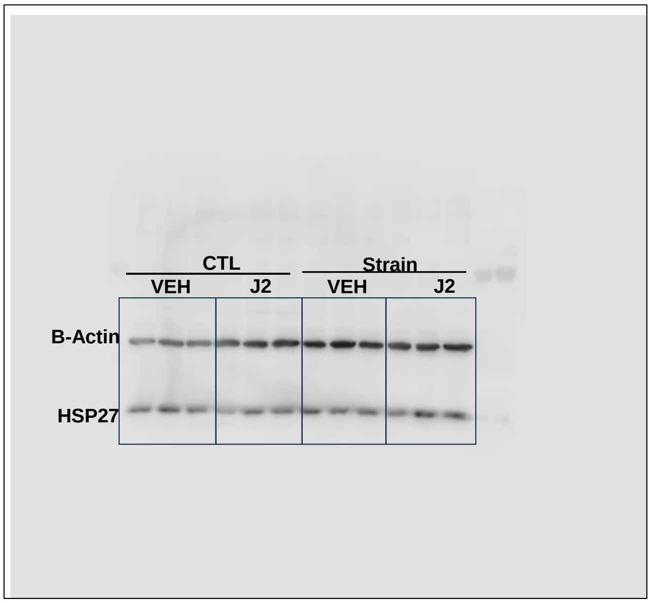

CTL
Strain
VEH
J2
VEH
J2
B-Actin
HSP27

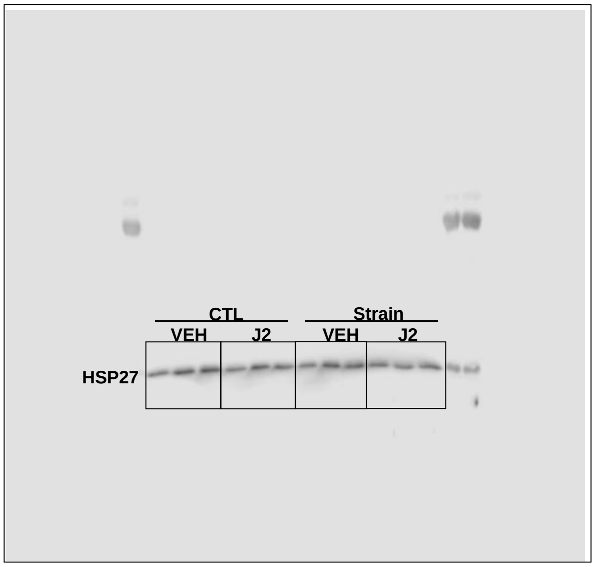

CTL
Strain
VEH
J2
VEH
J2
HSP27

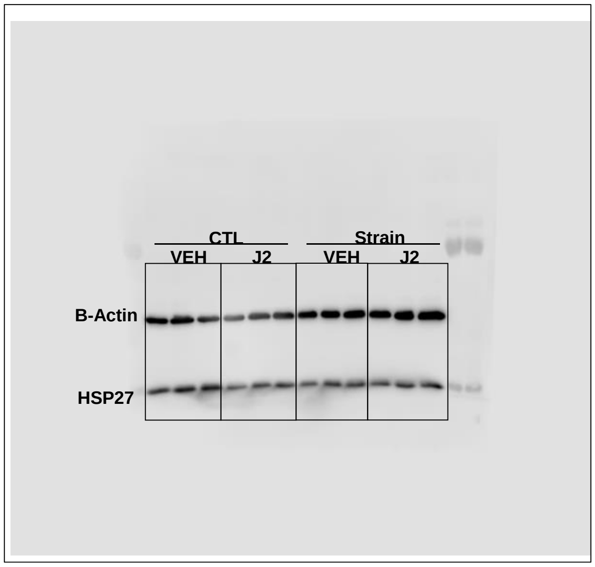

CTL
Strain
VEH
J2
VEH
J2
B-Actin
HSP27

Contrast Enhanced:

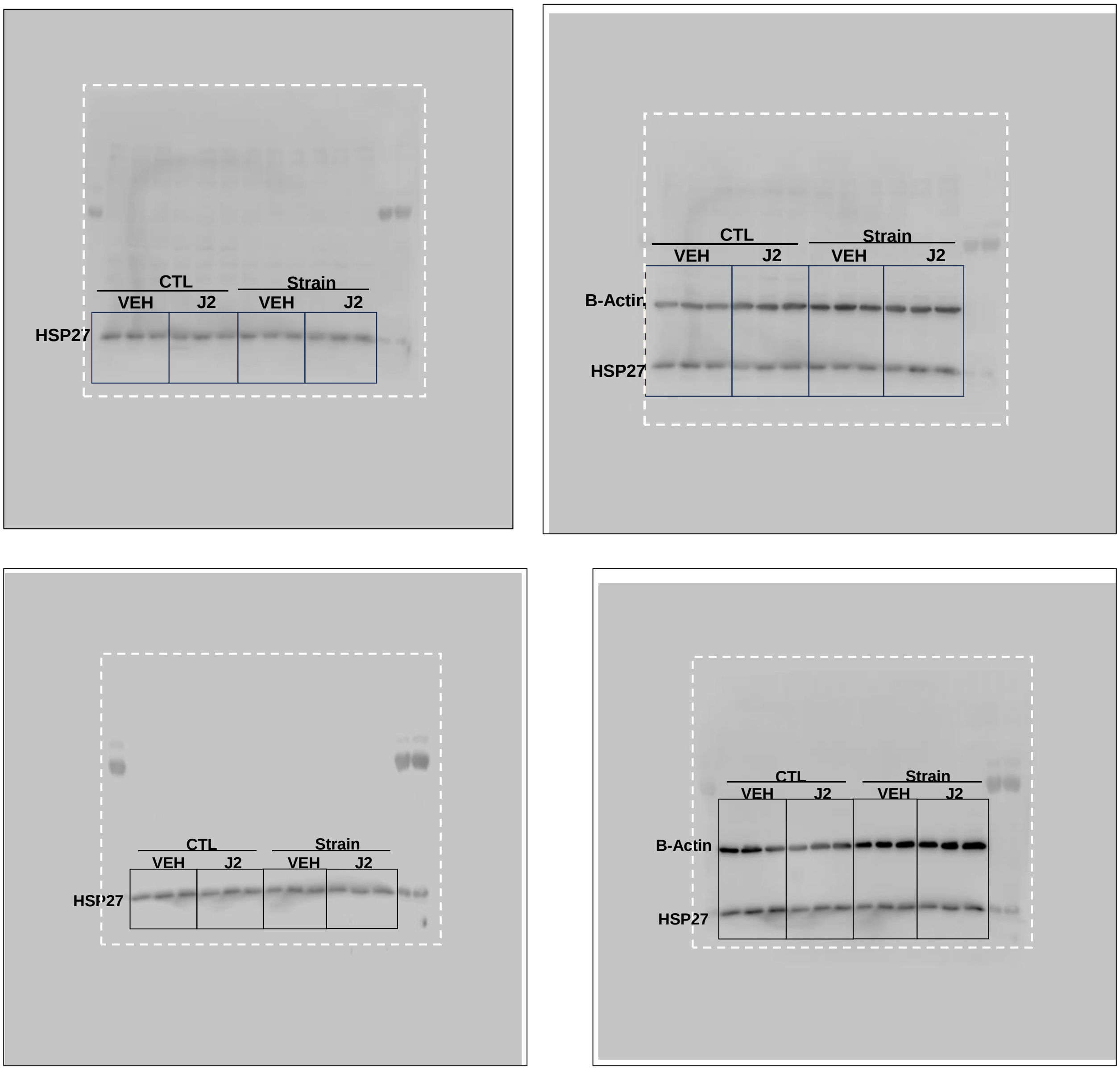

Figure S6. Uncropped Western blot images for SKOV-3 and SKOV-3.tr cells treated with strain and HSP27 Inhibitor. Data shown in Fig. 4A (SKOV-3, top) and B (SKOV-3.tr, bottom), total HSP27, and β-actin loading controls. All samples originated in the same experiment, and membranes were processed in parallel. Different exposure times are shown for the different proteins, due to inconsistencies with binding affinities for the different primary antibodies. Additional bands are internal controls or different experiments not included in this

manuscript. All images have been post-processed with the same techniques to ensure accurate quantification and comparisons. For clarity and completeness, the four images are fully uncropped and unmodified images from the Licor software, annotated to show bands of interest. The auto contrast on this system makes it difficult to see the edges of membranes in some cases, so we have provided contrast enhanced images (bottom four figures) with white dashed outlines to indicate membrane edges.

Unmodified:

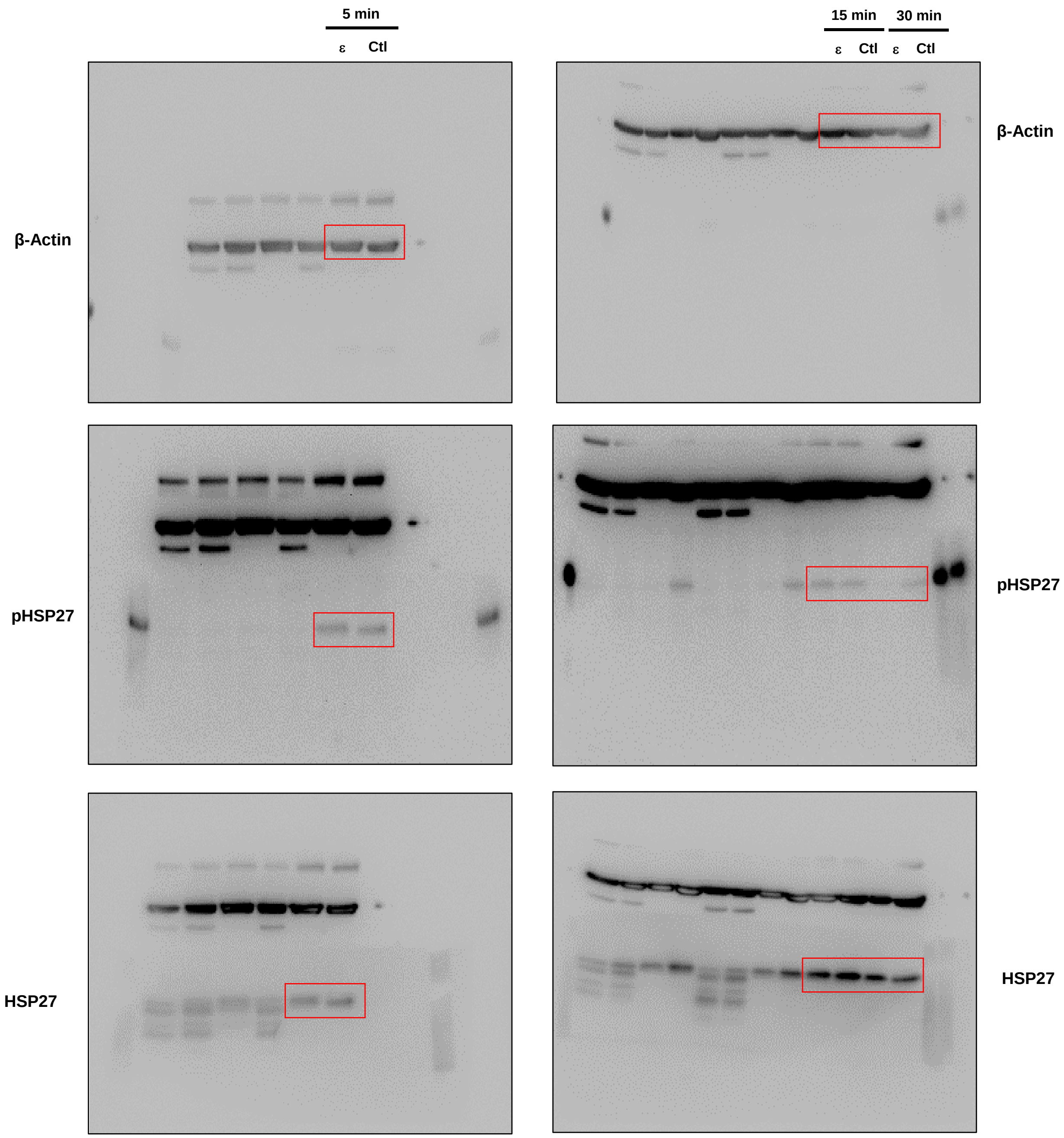
5 min
ε
Ctl
15 min
30 min
ε
Ctl
ε
Ctl
β-Actin
β-Actin
pHSP27
pHSP27
HSP27
HSP27

Modified:

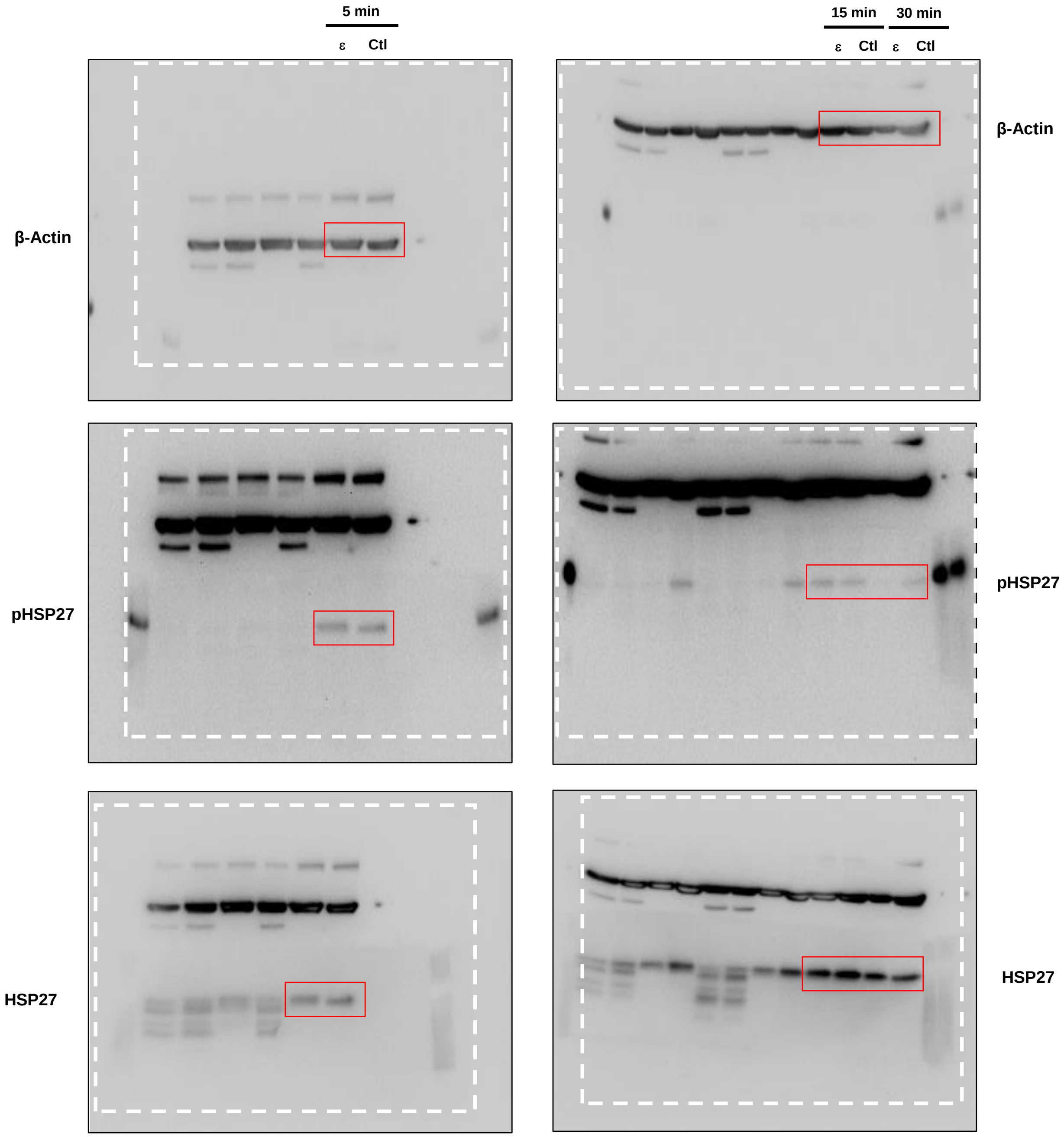

Figure S7. Uncropped Western blot images for OVCAR-8 cells treated with strain. Data shown in Fig. 9A, including both pHSP27, total HSP27, and β-actin loading controls, is highlighted with red boxes. All samples originated in the same experiment, and membranes were processed in parallel. Different exposure times are shown for the different proteins, due to

inconsistencies with binding affinities for the different primary antibodies. Additional bands are internal controls or different experiments not included in this manuscript. Total HSP27 was measured on the same membrane after processing with stripping solution; this is the reason why the image is not perfectly aligned with the β-actin and pHSP27 images. All images have been post-processed with the same techniques to ensure accurate quantification and comparisons. For clarity and completeness, the six images are fully uncropped and unmodified images from the Gel-Doc software, annotated to show bands of interest. The auto contrast on this system makes it difficult to see the edges of membranes in some cases, so we have provided contrast enhanced images (bottom six figures) with white dashed outlines to indicate membrane edges. The switch between Gel-Doc imaging and Licor imaging was based on replacing old equipment; all experiments directly comparing samples were completed on the same imager.
